# Nonlinear control of two-stage single-phase standalone photovoltaic system

**DOI:** 10.1371/journal.pone.0297612

**Published:** 2024-02-08

**Authors:** Adil Latif, Laiq Khan, Shahrukh Agha, Sidra Mumtaz, Jamshed Iqbal

**Affiliations:** 1 Department of Electrical and Computer Engineering, COMSATS University Islamabad, Abbottabad Campus, Abbottabad, KPK, Pakistan; 2 Department of Electrical and Computer Engineering, COMSATS University Islamabad, Islamabad, Pakistan; 3 School of Computer Science, Faculty of Science and Engineering, University of Hull, Hull, United Kingdom; J.C. Bose University of Science and Technology, YMCA, INDIA, INDIA

## Abstract

This paper presents a single-phase Photovoltaic (PV) inverter with its superior and robust control in a standalone mode. Initially, modeling and layout of the Buck-Boost DC-DC converter by adopting a non-linear Robust Integral Back-stepping controller (RIBSC) is provided. The controller makes use of a reference voltage generated through the regression plane so that the operating point corresponding to the maximum power point (MPP) could be achieved through the converter under changing climatic conditions. The other main purpose of the Buck-Boost converter is to act like a transformer and produce an increased voltage at the inverter input whenever desired. By not using a transformer makes the circuit size more compact and cost-effective. The proposed RIBSC is applied to an H-bridge inverter with an LC filter to produce the sinusoidal wave in the presence of variations in the output to minimize the difference between the output voltage and the reference voltage. Lyapunov stability criterion has been used to verify the stability and finite-time convergence of the overall system. The overall system is simulated in MATLAB/Simulink to test the system performance with different loads, varying climatic conditions and inverter reference voltages. The proposed methodology is compared with a back-stepping controller and Proportional Integral Derivative (PID) controller under rapidly varying climatic conditions. Results demonstrated that the proposed technique yielded a tracking time of 0.01s, a total harmonic distortion of 9.71% and a root means square error of 0.3998 in the case of resistive load thus showing superior control performance compared to the state-of-the-art control techniques.

## 1. Introduction

An independent system based on renewable power resources is one of the high-quality alternatives to meet the power demand in far-off and remote areas where the application grids are difficult to access or expensive [1]. Such systems are modeled to function irrespective of the application grid, as indicated by the name. A standalone device consists of various components that transform renewable energy sources including wind and Photovoltaic (PV) energy in a controlled and reliable way into electrical charges and electrical energy.

There are two types of existing PV systems: stand-alone [2], and grid-connected [3]. To connect the DC power supply to the grid at the PV level, the use of a converter as an interface is very important [4, 5]. This system is called a PV network connected to the grid. On the other hand, stand-alone PV systems include converting PV power into AC loads to be used at the user’s location. Power converters are necessary if solar-based PV modules and AC loads are to be interconnected. Such a power converter serves two main functions: First, it ensures that maximum power is generated continuously by the PV array irrespective of atmosphere, and load conditions. The second functionality is to alter the uniform voltage generated by the PV array into an AC voltage for AC load use. For example, a grid with stable repeatability, amplitude, and sinusoidal form should have identical performance and parameters for the AC output voltage. To attain these functions, several topologies are illustrated in [6, 7].

In this article, a two-phase-topology converter is being used. This requires an H-bridge inverter and buck-boost converter. The main purpose of the primary converter (buck-boost converter) is to allow the PV array to use the Maximum Power Point Tracking (MPPT) scheme to produce the maximum power [8]. Many algorithms can be used to adjust Maximum Power Point (MPP) correctly. Martin and Vazquez [9] used the back-stepping algorithm to achieve MPPT. Numerous researchers have discussed two extensive categories of MPPT technology: indirect MPP technology (such as open-circuit fractional voltage technology [10]), direct MPP technology (such as incremental conductivity [11, 12]) or Perturb and Observe (P & O) technologies. Motahhir et al. in [11] used the proposed incremental conductance to achieve a response time of 7ms to follow the appropriate power value with 97.53% efficiency. In [12], reinforcement times of 2.42ms and 2.17ms were achieved corresponding to the MPPT back-stepping control and integral back-stepping control respectively. In [13], efficiencies of 96%, 96.5%, 98.2%, and 99.1% were achieved using P & O technology, PI controller, Sliding Mode Control (SMC), neuro-fuzzy, group optimization, and back-stepping respectively. Our key goal is to obtain a shorter response time in the MPPT phase. P & O technology has the deterministic advantage, that is, the P-V curve rises on the left side of the MPP, and essentially drops on the right side of the MPP. The main drawback of this technique is that it always fluctuates around the MPP area. This problem can be reduced by making small movements around the MPP. The DC-DC converter has another defect of no output voltage regulation. Therefore, this fact must be brought into consideration as reported by Kaouane et al. in [14]. In [15], Chen et al. used various optimization techniques including Particle Swarm Optimization (PSO) to obtain the maximum power by designing an MPPT controller to enhance the efficiency of a PV inverter.

An inverter is part of the second stage of the proposed solution. To ensure the effective usage of Distributed Generation (DG) units, modeled inverters play the function of converting and adapting energy between a source and a load [16]. In these inverters, the conversion rule is to use a pulse width modulation (PWM) program to provide the load with a stable 220V (RMS) AC sinusoidal output voltage. Many inverters use electronic power switches on the output platform, for example, Insulated-Gate Bipolar Transistor(IGBTs) or Metal Oxide Semiconductor Field-effect Transistor (MOSFETs). The PWM program makes these inverters suitable for various electrical equipment [16]. Therefore, these inverters must have a minimum total harmonic distortion (THD), high efficiency, and rapid transient response. Many considerations have been made for the regulation of PWM inverters to ensure low THD, fast dynamic response, and constant frequency sinusoidal output voltage under various loads [17]. Proportional Integral Derivative (PID) control [18], sliding mode control [19], linear control [20], Lyapunov control [21], linear resonance control [22] and passive basic control [23] are the most well-recognized regulatory techniques. Zadeh et al. [24] proposed and evaluated three kinds of controllers: back-stepping, sliding mode, and fuzzy logic with the back-stepping controller demonstrating superior performance. Kolbasi and Seker [25] proposed a nonlinear inverter controller based on robust back-stepping. However, having more than two gains makes it harder for the controller to be efficient.

The control method based on the Lyapunov function proposed for a single-phase inverter in [26] achieved global balance with no steady-state error in the output voltage. Robust back-stepping control techniques for nonlinear systems are been developed in literature [27]. The designed controller provided a smoother controller movement for dynamic systems by providing an adjustment function to achieve the reference value. For an independent single-phase voltage source inverter, a control system based on droop-Lyapunov was proposed in [28]. The control system is determined by the dynamic model inverter that has evolved in the rotating body d-q. The dynamic version and the direct Lyapunov technology were used respectively to evaluate the reliability of the controller’s overall output under steady-state and dynamic operation. Therefore, a capacity curve (CC) was given to assess the most extreme positive and negative values of the inverter current component d-q.

Xue et al. [29] included a sketch of single-phase inverters generated for little-used generators. Yao et al. [30] suggested a single-phase, seamless transition of smart grid inverters between independent and grid-connected modes. Even though both independent power sources and power grid-related power sources can be attained through different circuit structures or current control plans [29, 30], the stability of the entire system cannot be demonstrated more comprehensively, and the new structure of the circuit system cannot be used it correctly in commercial products. It is a difficult task for scientists and researchers to get the maximum energy possible from the existing PV system [31]. Artificial neural networks, which employ intricate sets of qualitative heuristically developed rules and extensive training data sets, tend to increase the flexibility of the controller design. To lessen or prevent the overshoot in the load current and power, sliding-mode controllers have been proposed [32]. Additionally, these controllers play a crucial role in producing a reliable response, particularly when SMCs and ANN algorithms are combined [32].

In [33], Armghan et al. presented the modeling and design of a single-phase PV inverter with MPPT algorithm applied to the boost converter using back-stepping control in the standalone mode. The authors also made use of a DC-to-AC converter for AC loads. The response time to attain the exact value of the power of the PV array was around 1ms and the efficiency of the MPPT system was 99.93%. The response time of the inverter has a good form of the output voltage for electrical loads was 30ms at the beginning. Moreover, with an input voltage of the inverter greater than 311V, the response time of this controller was less than 15ms that ensuring the high robustness of the designed controller. Total Harmonic Distortion (THD) reported was about 0.78%. However, the response obtained was still oscillatory.

In [34], Arsalan et al. proposed a back-stepping-based non-linear controller for MPPT in a PV system. Authors showed the achievement of MPP with little oscillations under varying irradiance and temperature conditions. The MPP is achieved in 0.02 seconds under varying irradiance and 0.04 seconds under varying temperatures conditions. Also, the proposed methodology was shown to be superior, in terms of convergence speed and ripples compared to P & O and Fuzzy logic controller techniques which suffered from ripples and oscillations respectively.

In [35], Diouri et al. used integral action in the nonlinear back-stepping controller for MPPT of a PV system. A regression plane was used to generate the tracking peak power voltage. Under varying irradiance and temperature conditions, the tracking time was reported to be 2ms with the converter efficiency of 95%. However, an overshoot of 13.8V (4.7% of steady-state value) was observed in the response under varying irradiance and a small overshoot of 7.44V (2.6% of steady-state value) was observed under varying temperature conditions. The proposed technique was also compared with P & O and PID methods and is shown to be robust in comparison. Also, the proposed technique was compared with back-stepping and Fuzzy logic controller methods. Back-stepping method has a small steady error under varying irradiance and temperature conditions whereas the Fuzzy logic controller showed large oscillatory behavior around MPP.

In [36], Ali et al. proposed a nonlinear robust integral back-stepping-based MPPT control for a stand-alone PV system. A comparison was made with back-stepping, integral back-stepping, PID and P & O methods under three different operating conditions, including varying irradiance, temperature, and fault injections. A significant steady-state error and overshoot were reported to exist in the back-stepping (B) and integral back-stepping (IB) techniques, respectively. In comparison the proposed methodology of [36] achieves a tracking time of 0.02 seconds with 98% efficiency of the PV array. The proposed technique is also compared with PID and P & O methods and is shown to be robust with minimum chattering in comparison.

In [37] Khan et al. suggested a radial basis function (RBF) neural network-based back-stepping terminal sliding mode MPPT control technique for a stand-alone PV systems. The proposed methodology was reported to have a reduced chattering effect, improved transient response, and faster convergence, with a PV array efficiency of 98.74% when compared to the benchmarks of P & O, PID, and back-stepping controllers. From the above, it can be inferred that though the proposed methodologies have addressed the aforementioned issues under different conditions, there are still some robustness and oscillatory behavior issues, which are undesirable. For instance, the back-stepping technique is highly efficient but not robust in varying conditions.

In [38], an experimental estimation of a hybrid adaptive neuro-fuzzy inference system–particle swarm optimization (ANFIS–PSO) based MPPT for PV grid integration under fluctuating sun irradiance was presented. The method aims to achieve fast and maximal PV power with zero oscillation tracking. A space vector modulation hysteresis current controller was utilized to implement an inverter control strategy to obtain high-quality inverter current by accurately tracking the reference sine-shaped current. In [39], an extensive practical investigation of fuzzy particle swarm optimization (FPSO) based MPPT for grid-integrated PV system under variable operating conditions with anti-islanding protection was presented. Different conditions, such as varying sun irradiance, partial shadow, and loading conditions have been considered for the verification of the proposed methodology. Experimental implementation and verification of the proposed system, along with high tracking efficiency, were reported. In [40], an adaptive Takagi Sukeno (TS)-fuzzy model-based radial basis function (RBF) neural network learning for grid-integrated PV applications was presented. Rapid and accurate PV power tracking is reported to have been achieved under varying solar irradiance. Keeping this in view, the significant contributions in this work are as follows:

1. A robust controller is proposed that has a fast convergence time with negligible oscillations.
2. A PV array system, comprising a DC-DC buck-boost converter and an inverter, is suggested supplying maximum power achieved to AC loads under varying irradiance and temperature conditions. Additionally, the inverter has a minimum total harmonic distortion.
3. The proposed controller is compared with state-of-the-art benchmarks, namely PID and back-stepping controllers, and is demonstrated to exhibit faster convergence time, reduced steady-state error, fewer oscillations and ripples, faster rise times, and low settling times. Various performance metrics, such as Integral Time Square Error (ITSE), Integral Time Absolute Error (ITAE), Integral Square Error (ISE), and Integral Absolute Error (IAE), have been employed to showcase the superiority of the proposed controller.
4. The proposed technique is evaluated in two different scenarios: a) Steady-state performance of the inverter output voltage with different loads b) Transient performance of the inverter output voltage when the reference amplitude changes in steps. The execution of the proposed technique is analyzed and compared with the back-stepping and PID controllers.
5. The proposed scheme is highly efficient because it can rapidly adapt to sudden changes in climatic conditions, including temperature fluctuations, variations in sunlight, or both. Regarding voltage stability, the system can consistently maintain a voltage of 220V and a stable frequency of 50Hz under changing loads.

The rest of the paper is organized into five main sections i.e. System overview and model description is presented in section 2. In addition, the non-inverting buck-boost converter and H-bridge inverter along with the average state-space layout is presented. Section 3 introduces the PV control system architecture designed to achieve full power and transform the DC voltage to the AC voltage. In section 4, the design and simulation of the proposed technique is explained, and the result of the proposed technique comparison are discussed and analyzed under different load conditions and step changes in reference amplitude. Section 5 provides the paper’s conclusion, along with suggestions for future work.

## 2. System overview and model description

**Fig 1:**
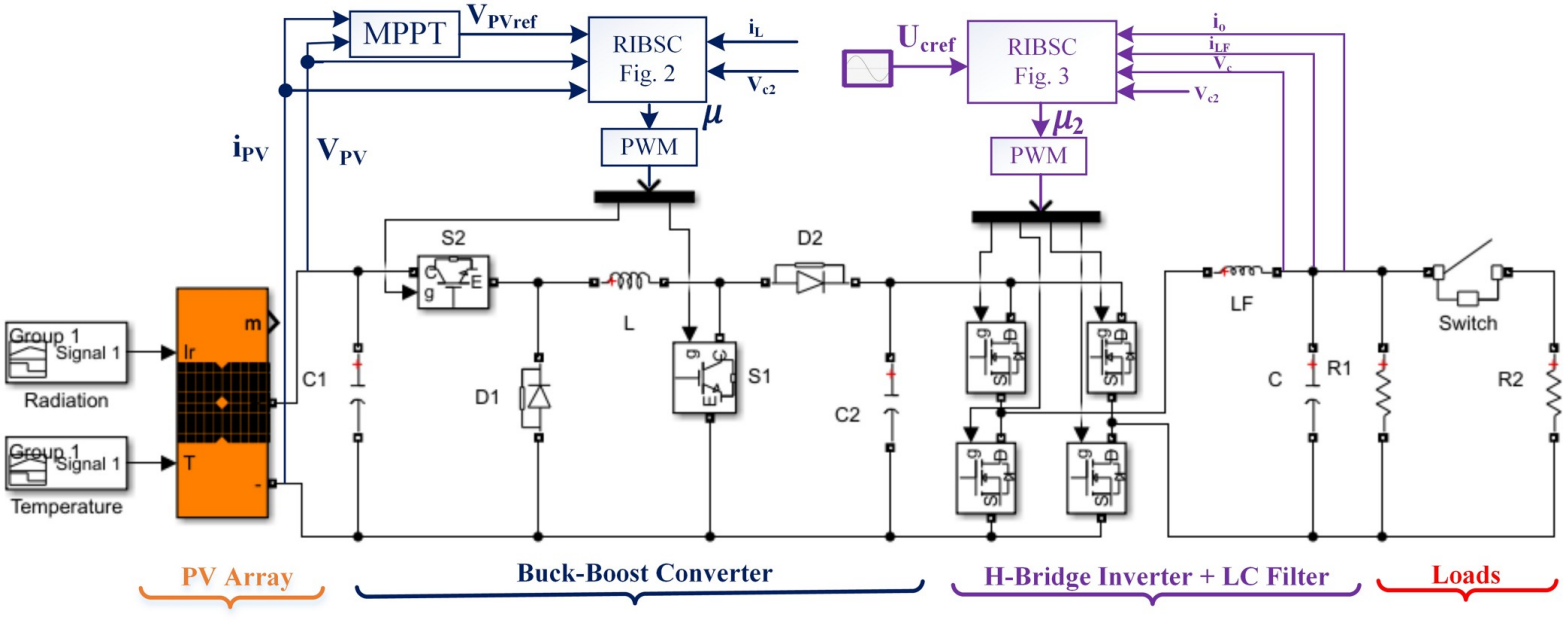
A standalone PV system model.

**Fig 2:**
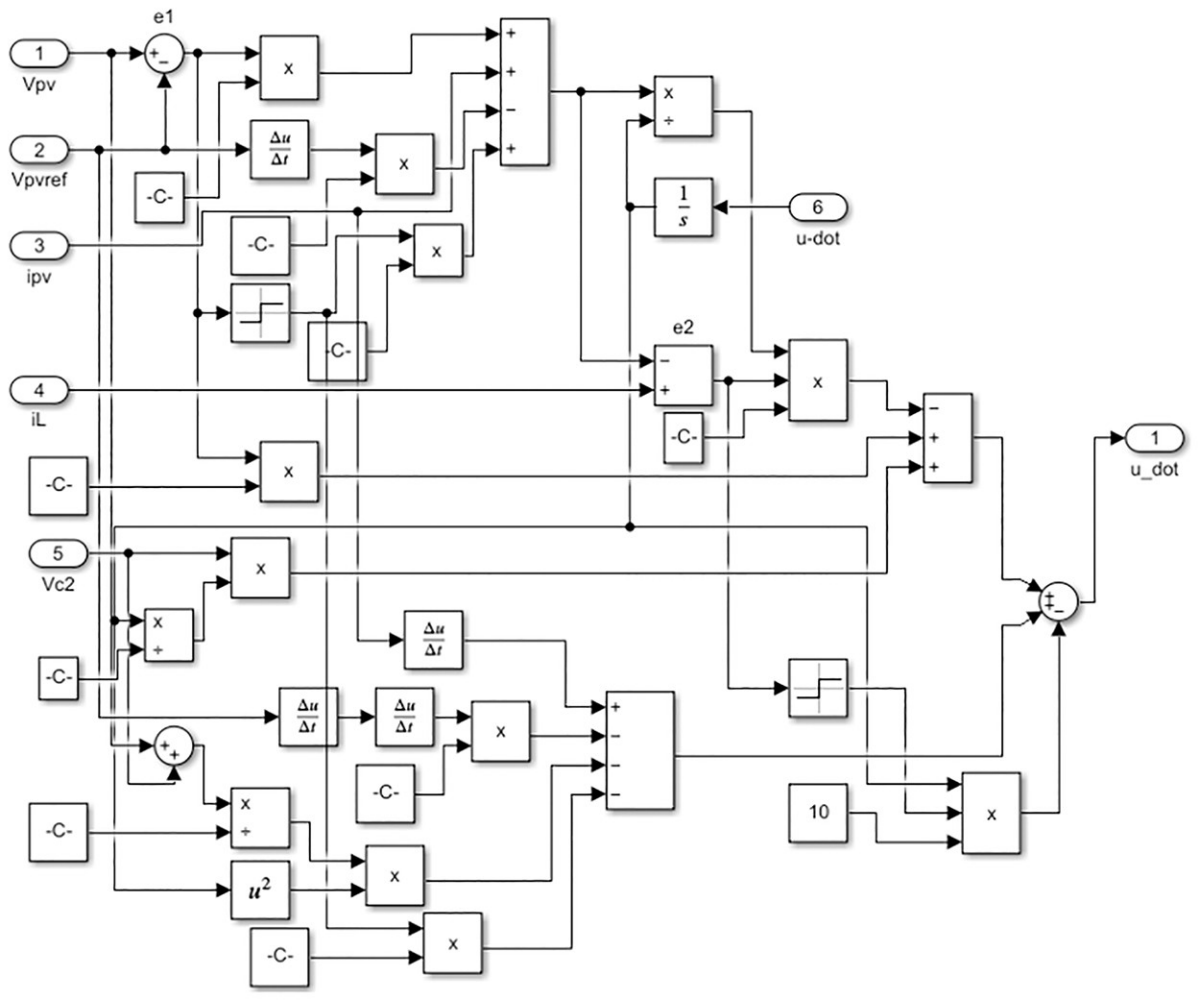
Internal blocks of RIBSC for converter.

**Fig 3:**
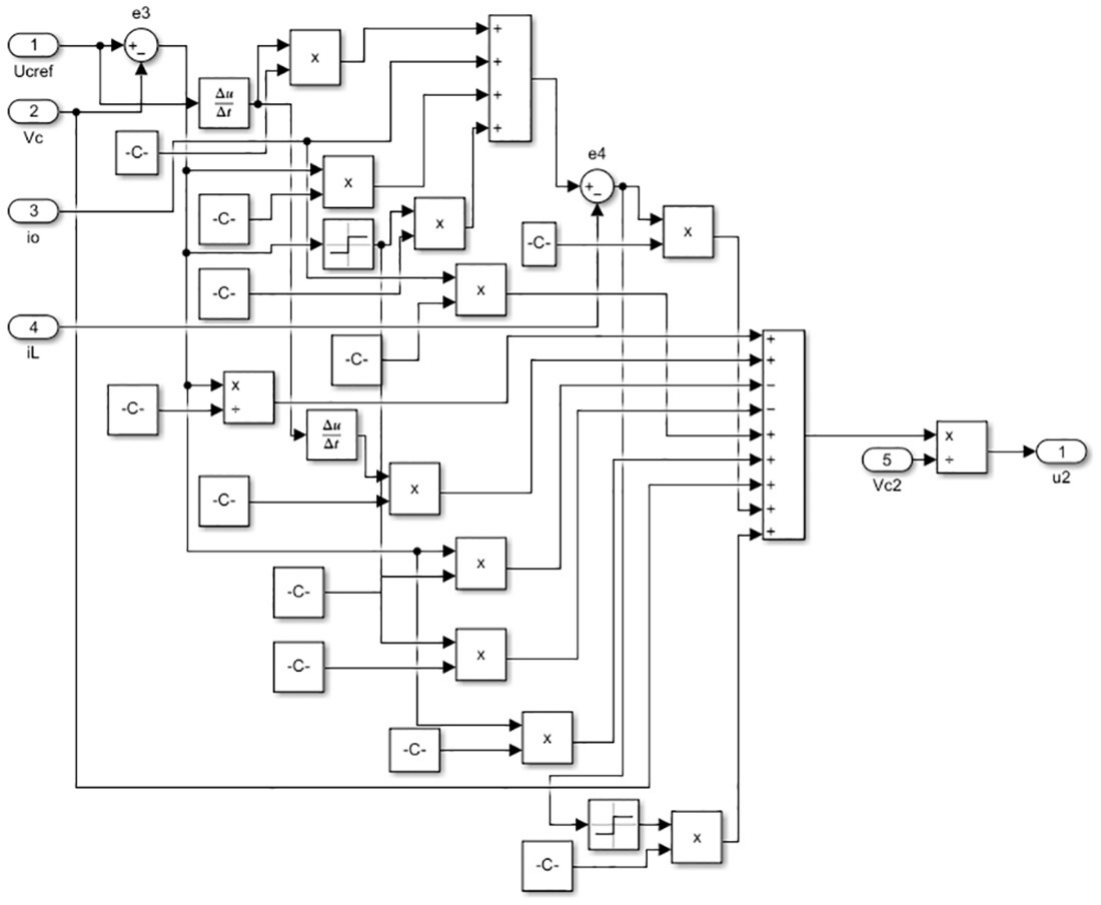
Internal blocks of RIBSC for inverter.

A typical model of a PV system takes into consideration economic operation and is usually comprised of a DC-AC inverter, an MPPT controller strategy, a DC-DC converter, and PV modules. The PV array and the AC loads are connected by two power electronic converters in the system. To achieve maximum power delivery to the loads and ensure proper DC to AC power conversion, both converters are controlled by a Robust Integral Back-stepping Controller (RIBSC). The first converter, using a simple MPPT algorithm to tracks the maximum electrical energy produced by the PV array under various temperatures and irradiance levels. To compel the PV array to supply this voltage, it generates the reference voltage for the controller. The second converter, equipped with an LC filter, is responsible for converting continuous voltage to alternating voltage while reducing harmonic distortion and maintaining an acceptable amplitude and frequency stability across various resistive load values. The block diagram of the simulation is depicted in Fig 1. The internal blocks of RIBSC for converter and inverter are depicted in Figs 2 and 3, respectively.

### 2.1 Mathematical modeling of PV system

**Fig 4:**
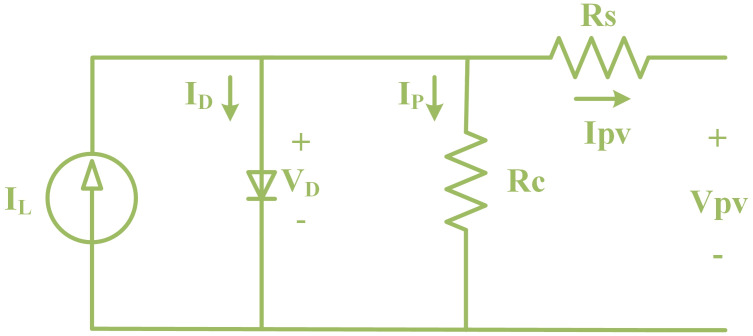
PV array equivalent model.

The PV cell is configured so that it takes solar irradiance as input and generates electrical energy as output [41]. Typically, electrical equivalent models of PV cells are presented as either single-diode models [42] or two-diode models [43]. The two-diode model, being more complex, requires additional parameters to design a precise model. This research opts for a single-diode version for its simplicity. The PV cell resembles a diode with a p-n junction, which converts photons into electricity. As displayed in Fig 2, a diode model is composed of a diode *D*, current source *I*_*L*_, shunt resistance *R*_*C*_, and series resistance *R*_*S*_. To determine the corresponding circuit parameters, PV voltage, current, and power curves must be observed under standard measuring conditions. Given that the value of *R*_*C*_ is relatively high and the value of *R*_*S*_ is low, these two parameters can be neglected to simplify the analysis [44]. The following equations, with the application of Kirchhoff’s Current Law (KCL), describes the equivalent circuit shown in Fig 4.

$$\begin{array}{c} I_{PV}=I_{L}-I_{D}-I_{P} \end{array}$$ (1)

where the photo-current generated by the PV cell is denoted as *I*_*L*_, and it depends on the temperature (T), solar radiation, and diode current *I*_*D*_. Similarly, *I*_*P*_ represents the shunt current, and *I*_*PV*_ is the output current in amperes of the PV cell. When a number of PV cells are connected in series and parallel, they form a PV module or panel. Eq (2) illustrates the PV module’s output current *I*_*pv*_ to the output voltage *V*_*PV*_ when *N*_*S*_ and *N*_*P*_ cells are connected in series and parallel, respectively [41].

$$\begin{array}{c} I_{pv}=N_{p}I_{L}-N_{p}I_{D}[\text{exp}\;\frac{qV_{pv}}{N_{S}AkT}-1] \end{array}$$ (2)

where *V*_*PV*_ is the output voltage of the PV module in volts, *N*_*P*_ and *N*_*S*_ represents the PV cells connected in parallel and series, respectively. The ideality factor of a diode is denoted as *A*, *T* is the temperature in Kelvin, *k* is the Boltzmann constant in *J*/*K* and the electron charge is *q* in Coulombs. The reverse saturation current of the PV module, *I*_*D*_, is given by:

$$\begin{array}{c} I_{D}=I_{Dr}{\left(\frac{T}{T_{r}}\right)}^{3}\;\text{exp}\;(\frac{qE_{g}}{kA}[\frac{1}{T_{r}}-\frac{1}{T}]) \end{array}$$ (3)

where Eg = 1.1 eV is the semiconductor band gap, and the reference temperature of the cells is *T*_*r*_. Therefore, at *T*_*r*_, the reverse saturation current *I*_*Dr*_ is specified by the following equation.

$$\begin{array}{c} I_{Dr}=\frac{I_{scr}}{\text{exp}\;\left(\frac{qV_{oc}}{N_{S}AkT}\right)-1} \end{array}$$ (4)

Among them, *V*_*OC*_ is the open-circuit voltage, and *I*_*scr*_ is the short-circuit current of the PV module at the reference temperature *T*_*r*_. The dependence of *I*_*L*_ on temperature and solar irradiance is described by the following equations.

$$\begin{array}{c} I_{L}=[I_{scr}+K_{i}\left(T-T_{r}\right)]\frac{E}{100} \end{array}$$ (5)

where *K*_*i*_[*A*/*K*] is the temperature coefficient of *I*_*SCr*_.

A mathematical description of the output power of a PV module, derived from Eq (2), is coined below.


$$\begin{array}{c} P_{pv}=I_{pv}V_{pv} \end{array}$$ (6)

$$\begin{array}{c} P_{pv}=N_{p}I_{ph}V_{pv}-N_{p}I_{D}V_{pv}[\text{exp}\;(\frac{qV_{pv}}{N_{S}AkT})-1] \end{array}$$ (7)

**Fig 5:**
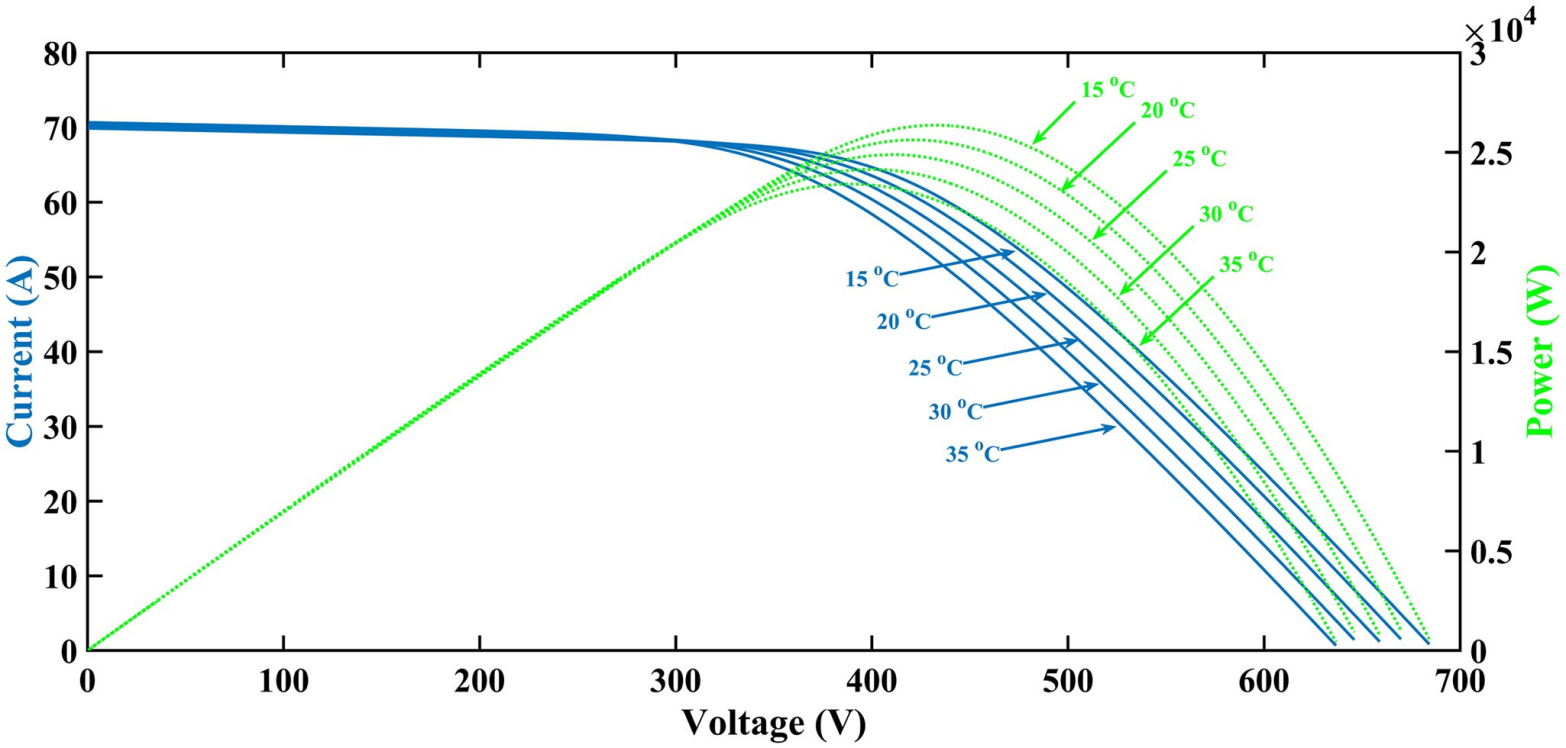
I-V and P-V characteristics curves at rising temperature.

**Fig 6:**
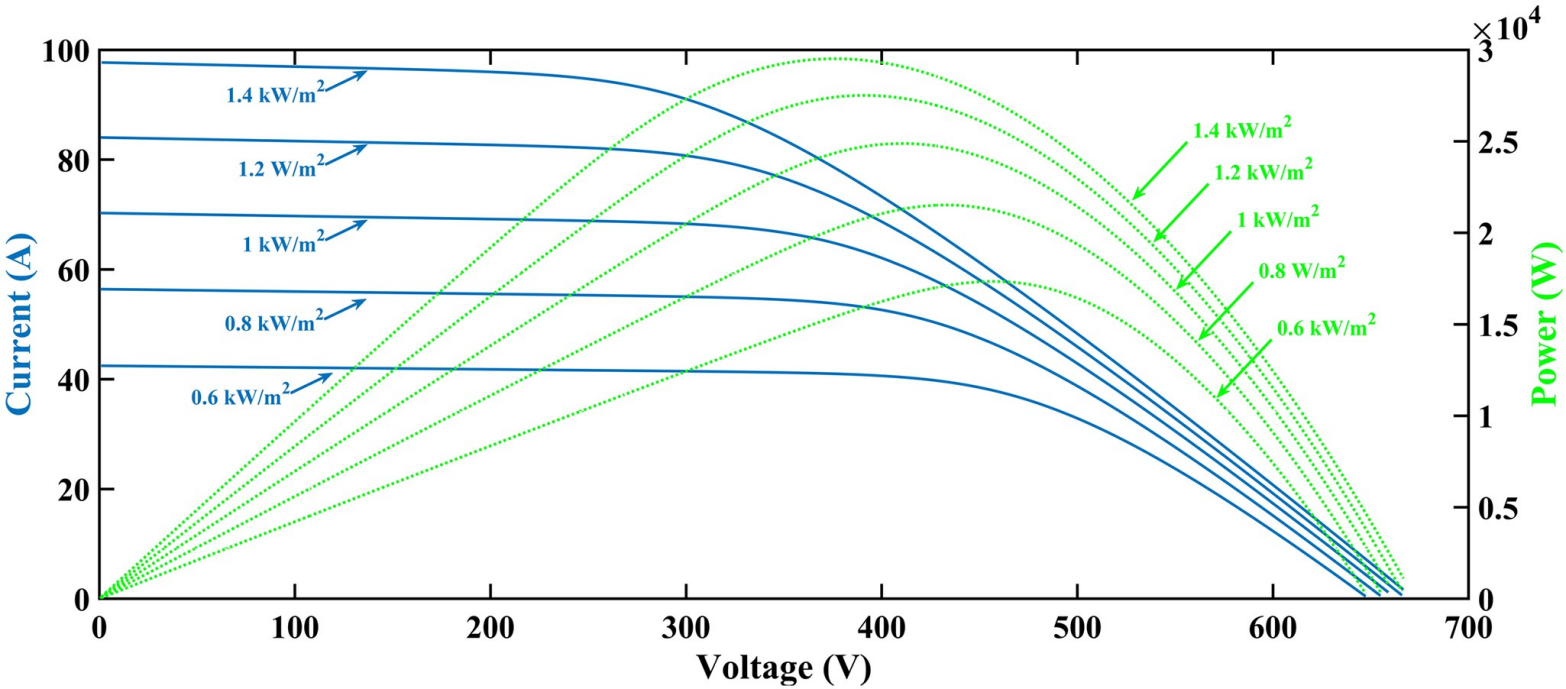
I-V and P-V characteristics curves at rising irradiance.

The PV array considered in this article is composed of four strings that can be connected in parallel. Each string includes series-connected four modules. Thus, the overall amount of PV modules utilized is sixteen. Each module has an intensity of 1555W. Therefore, the maximum power provided by the system is 16 × 1555 = 24,880W. Figs 5 and 6 shows the PV array’s (I-V) and (P-V) characteristics for various temperature (T) and irradiance values, respectively.

### 2.2 Mathematical modeling of non-inverting DC to DC buck-boost converter

**Fig 7:**
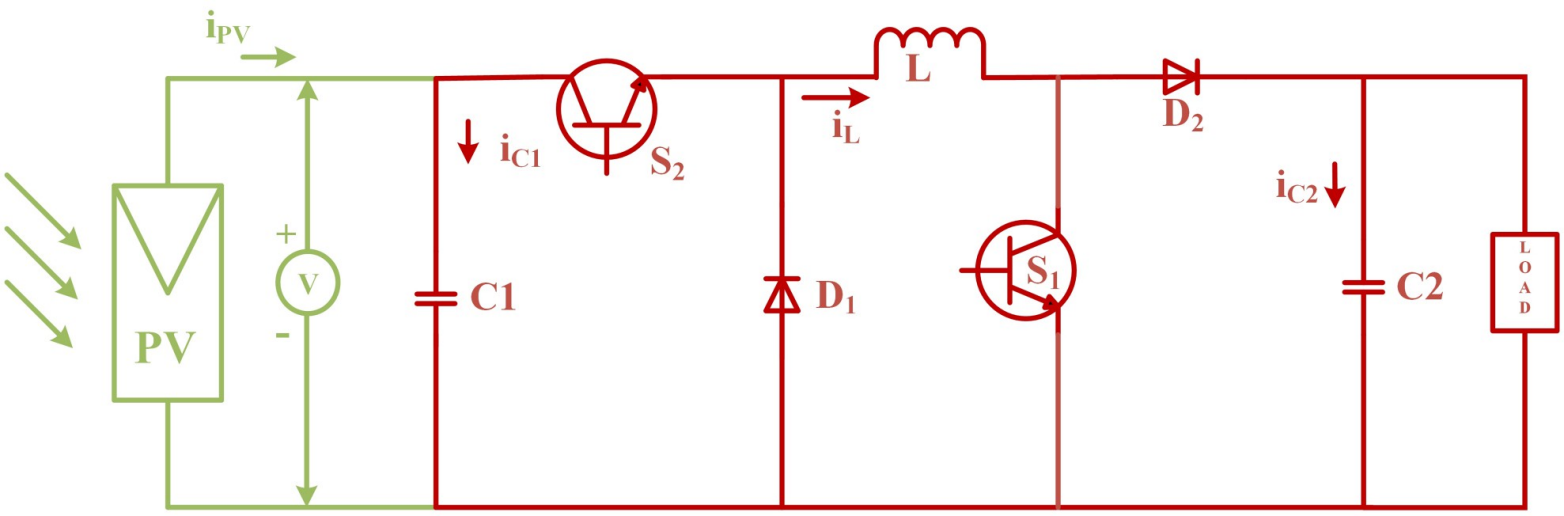
Non-inverting DC-DC buck-boost circuit topology.

The output voltage *V*_*PV*_ of the PV array can be raised or lowered using a designed converter to match the reference voltage *V*_*PVref*_. The non-inverting buck-boost converter is responsible for tracking the maximum electrical energy produced by the PV array at various temperatures and irradiance levels to meet the reference voltage for the back-stepping controller. Fig 7 demonstrates the in-phase DC-DC buck-boost converter circuit topology. The output voltage can be regulated by changing the converter’s duty cycle to maximize output power through designed controllers. The converter operates in continuous conduction mode (CCM) while assuming ideal semiconductor switches and diodes [45].

This converter is commonly used in applications that require low cost and compact component size. The converter is continuously controlled through its changing period *T*, where: *T* = *t*_*on*_ + *t*_*off*_, and *μ* = *t*_*on*_/*T* is defined as the converter duty ratio. Input voltage ripples of the converter are limited by the input capacitor *C*_1_, and output voltage ripples are restricted by *C*_2_. The switching operation involves two periods. First, diodes *D*_1_ and *D*_2_ are in reverse bias, while switches *S*_1_ and *S*_2_ are both in conduction mode. In the second period, switches *S*_1_ and *S*_2_ are turned OFF, and *D*_1_ and *D*_2_ are forward biased. The state-space equation for mode 1, when switches are in conduction mode and the diodes are reverse biased, can be represented in matrix form as follows:

$$\begin{array}{c} \left[\begin{array}{c} \frac{d}{dt}V_{PV}\\ \frac{d}{dt}i_{L}\\ \frac{d}{dt}V_{c2} \end{array}]=[\begin{array}{ccc} 0 & -\frac{1}{C_{1}} & 0\\ \frac{1}{L} & 0 & 0\\ 0 & 0 & -\frac{1}{R_{L}C_{2}} \end{array}][\begin{array}{c} V_{PV}\\ i_{L}\\ V_{c2} \end{array}]+[\begin{array}{c} \frac{i_{PV}}{C_{1}}\\ 0\\ 0 \end{array}\right] \end{array}$$ (8)

The state-space equation for mode 2, when diodes are forward-biased and switches are OFF. The equation in matrix form is given as:

$$\begin{array}{c} \left[\begin{array}{c} \frac{d}{dt}V_{PV}\\ \frac{d}{dt}i_{L}\\ \frac{d}{dt}V_{c2} \end{array}]=[\begin{array}{ccc} 0 & 0 & 0\\ 0 & 0 & -\frac{1}{L}\\ 0 & \frac{1}{C_{2}} & -\frac{1}{R_{L}C_{2}} \end{array}][\begin{array}{c} V_{PV}\\ i_{L}\\ V_{c2} \end{array}]+[\begin{array}{c} \frac{i_{PV}}{C_{1}}\\ 0\\ 0 \end{array}\right] \end{array}$$ (9)

Now, the average model of the non-inverting DC-DC buck-boost converter in the form of a vector matrix is as follows, based on volt-second balance and capacitor charge level:

$$\begin{array}{c} \left[\begin{array}{c} \frac{d}{dt}V_{PV}\\ \frac{d}{dt}i_{L}\\ \frac{d}{dt}V_{c2} \end{array}]=[\begin{array}{ccc} 0 & -\frac{\mu }{C_{1}} & 0\\ \frac{\mu }{L} & 0 & \frac{\mu }{L}-\frac{1}{L}\\ 0 & \frac{1}{C_{2}} & -\frac{1}{R_{L}C_{2}} \end{array}][\begin{array}{c} V_{PV}\\ i_{L}\\ V_{c2} \end{array}]+[\begin{array}{c} \frac{i_{PV}}{C_{1}}\\ 0\\ 0 \end{array}\right] \end{array}$$ (10)

Considering *x*_1_, *x*_2_, and *x*_3_ as the average values of *V*_*PV*_, *i*_*L*_, and *V*_*C*2_, respectively. Under the assumption, the state representation is as follows:

$$\begin{array}{c} \left[\begin{array}{c} {\dot{x}}_{1}\\ {\dot{x}}_{2}\\ {\dot{x}}_{3} \end{array}]=[\begin{array}{ccc} 0 & -\frac{\mu }{C_{1}} & 0\\ \frac{\mu }{L} & 0 & \frac{\mu }{L}-\frac{1}{L}\\ 0 & \frac{1}{C_{2}}-\frac{\mu }{C_{2}} & -\frac{1}{R_{L}C_{2}} \end{array}][\begin{array}{c} x_{1}\\ x_{2}\\ x_{3} \end{array}]+[\begin{array}{c} \frac{i_{PV}}{C_{1}}\\ 0\\ 0 \end{array}\right] \end{array}$$ (11)

Interpretation from matrix shape to equation form is as follows:

$$\begin{array}{c} {\dot{x}}_{1}=\frac{i_{PV}}{C_{1}}-\frac{\mu x_{2}}{C_{1}} \end{array}$$ (12)

$$\begin{array}{c} {\dot{x}}_{2}=-\frac{x_{3}}{L}+\mu \frac{x_{1}+x_{3}}{L} \end{array}$$ (13)

$$\begin{array}{c} {\dot{x}}_{3}=-\frac{x_{2}}{C_{2}}-\frac{x_{3}}{R_{L}C_{2}}-\frac{\mu x_{2}}{C_{2}} \end{array}$$ (14)

A buck-boost converter model that does not invert is required. The Converter’s voltage transformation ratio is:

$$\begin{array}{c} \frac{V_{c_{2}}}{V_{PV}}=\frac{\mu }{1-\mu } \end{array}$$ (15)

The reflected input impedance on an ideal transfer of power is provided by [33],

$$\begin{array}{c} R_{out}=R_{in} \end{array}$$ (16)

where,

$$\begin{array}{c} R_{in}={\left(\frac{1-\mu }{\mu }\right)}^{2}R_{L} \end{array}$$ (17)

and,

$$\begin{array}{c} R_{out}=\frac{V_{mpp}^{2}}{P_{mpp}} \end{array}$$ (18)

where *V*_*mpp*_ is the voltage corresponding to the MPP and *P*_*mpp*_ is the corresponding maximum power.

### 2.3 Mathematical modeling of single-phase H-bridge inverter

**Fig 8:**
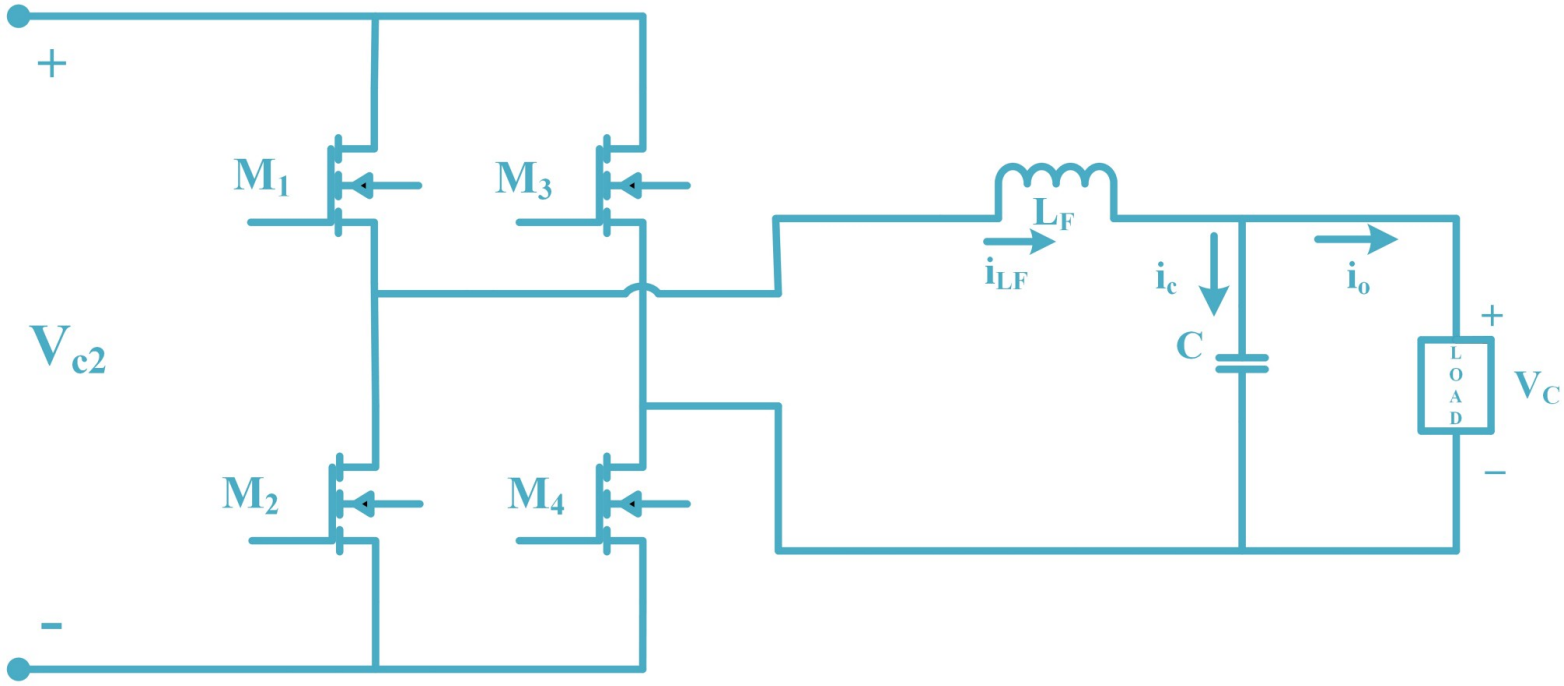
Basic diagram of a single-phase H-bridge inverter with LC filter.

The H-bridge inverter with an LC filter has the capability to convert direct voltage to alternating voltage with minimal harmonic distortion and good frequency and amplitude stability across a range of resistive load values. Fig 8 displays a PWM inverter cascaded with the LC filter in standalone mode with a RIBSC. The inverter device consists of two basic elements: the power components and the device control unit [30].

The power component includes the following:

1. An H-bridge inverter typically composed of four electrical MOSFETs.
2. The LC filter, essential for obtaining a sine wave with appropriate recursion and minimizing output voltage distortion.

The second part consists of a controller, which is RIBSC. The RIBSC control law is derived in the next section. *V*_*c*2_ is the DC voltage, *V*_*C*_ is the output voltage after filtering, *I*_*LF*_ is the current of the inductor *L*_*F*_, and *i*_*o*_ is the load current. The switching frequency applied to the electronic switch, which is 20 kHz, must be greater than the system frequency, which is 50 Hz, to obtain a reasonable inverter output voltage. Therefore, the Root Mean Square (RMS) of current and voltage are used. There are two modes for switching.

The state-space equation for when switches M1 and M4 are ON and M2 and M3 are OFF is as follows:

$$\begin{array}{c} \left[\begin{array}{c} \frac{d}{dt}V_{c}\\ \frac{d}{dt}i_{L_{F}}\\ \frac{d}{dt}V_{c_{2}} \end{array}]=[\begin{array}{ccc} 0 & \frac{1}{C} & 0\\ -\frac{1}{L_{F}} & 0 & \frac{1}{L_{F}}\\ 0 & 0 & 0 \end{array}][\begin{array}{c} V_{c}\\ i_{L_{F}}\\ V_{c_{2}} \end{array}]+[\begin{array}{c} -\frac{i_{o}}{C}\\ 0\\ 0 \end{array}\right] \end{array}$$ (19)

The state-space equation for when switches M1 and M4 are OFF and M2 and M3 are ON is as follows:

$$\begin{array}{c} \left[\begin{array}{c} \frac{d}{dt}V_{c}\\ \frac{d}{dt}i_{L_{F}}\\ \frac{d}{dt}V_{c_{2}} \end{array}]=[\begin{array}{ccc} 0 & \frac{1}{C} & 0\\ -\frac{1}{L_{F}} & 0 & -\frac{1}{L_{F}}\\ 0 & 0 & 0 \end{array}][\begin{array}{c} V_{c}\\ i_{L_{F}}\\ V_{c_{2}} \end{array}]+[\begin{array}{c} -\frac{i_{o}}{C}\\ 0\\ 0 \end{array}\right] \end{array}$$ (20)

Now, the average model for an H-bridge single-phase inverter in vector-matrix form is as:

$$\begin{array}{c} \left[\begin{array}{c} \frac{d}{dt}V_{c}\\ \frac{d}{dt}i_{L_{F}}\\ \frac{d}{dt}V_{c_{2}} \end{array}]=[\begin{array}{ccc} 0 & \frac{1}{C} & 0\\ -\frac{1}{L_{F}} & 0 & \frac{\mu_{2}}{L_{F}}\\ 0 & 0 & 0 \end{array}][\begin{array}{c} V_{c}\\ i_{L_{F}}\\ V_{c_{2}} \end{array}]+[\begin{array}{c} -\frac{i_{o}}{C}\\ 0\\ 0 \end{array}\right] \end{array}$$ (21)

Taking into account the average value of *V*_*c*_, $i_{L_{F}}$, and $V_{c_{2}}$, represented as *x*_1_, *x*_2_, and *x*_3_. Additionally, *μ*_2_ is the inverter control law.

$$\begin{array}{c} \left[\begin{array}{c} {\dot{x}}_{1}\\ {\dot{x}}_{2}\\ {\dot{x}}_{3} \end{array}]=[\begin{array}{ccc} 0 & \frac{1}{C} & 0\\ -\frac{1}{L_{F}} & 0 & \frac{\mu_{2}}{L_{F}}\\ 0 & 0 & 0 \end{array}][\begin{array}{c} x_{1}\\ x_{2}\\ x_{3} \end{array}]+[\begin{array}{c} -\frac{i_{o}}{C}\\ 0\\ 0 \end{array}\right] \end{array}$$ (22)

Representation from matrix shape to equation form is as follows:

$$\begin{array}{c} {\dot{x}}_{1}=\frac{x_{2}}{C}-\frac{i_{o}}{C} \end{array}$$ (23)

$$\begin{array}{c} {\dot{x}}_{2}=-\frac{x_{1}}{L_{F}}+\frac{x_{3}\mu_{2}}{L_{F}} \end{array}$$ (24)

$$\begin{array}{c} {\dot{x}}_{3}=0 \end{array}$$ (25)

### 2.4 Reference voltage generation by regression plane

**Fig 9:**
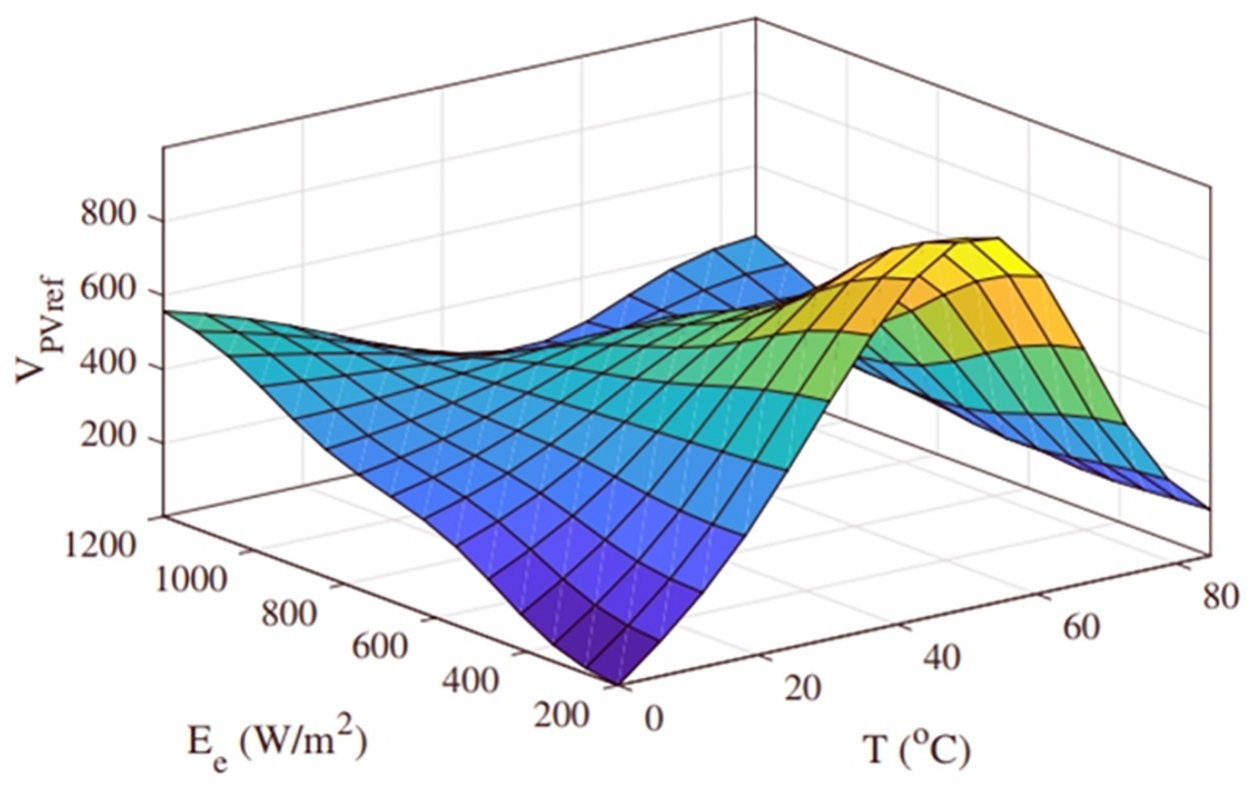
Regression plane.

The PV characteristic curve changes with each specific estimation of irradiance and temperature. Even a slight change in either of them results in a new curve, subsequently altering the MPP, as depicted in Figs 5 and 6 [34]. By consistently changing temperature from 15ºC to 35ºC while maintaining a constant irradiance level of 1000*W*/*m*^2^, different MPPs have been recorded. Furthermore, by using linear regression at a constant temperature of 25 degrees Celsius, additional data points have been collected for irradiance levels ranging from 600*W*/*m*^2^ to 1400*W*/*m*^2^, resulting in a three-dimensional plane, as shown in Fig 9. Subsequently, for any temperature and irradiance level, a reference voltage (*V*_*PVref*_) can be calculated as follows.

$$\begin{array}{c} V_{PVref}=579.81-1.9899*T-0.12146*I \end{array}$$ (26)

Where *I* is the irradiance and *T* is the temperature.

## 3. Proposed control algorithms

### 3.1 Control law for buck-boost converter using robust integral back-stepping controller

The primary objective of the control law is to attain MPP at the PV panel’s output by continuously adjusting the duty cycle (*μ*) of the buck-boost converter. This goal is accomplished through a robust integral back-stepping control law, which drives the converter’s output to track the reference (*V*_*PVref*_) generated by the regression plane using linear interpolation. To design and implement a robust integral back-stepping control method [46] and to stabilize the buck-boost control action at the origin region (zero error), we need to define an error (*e*_1_). This error is defined to enforce the input PV voltage *V*_*PV*_ to follow the regression plane’s reference voltage (*V*_*PVref*_) to track the MPP, as shown in Eq (27).

$$\begin{array}{c} e_{1}=x_{1}-V_{PVref} \end{array}$$ (27)

where (*V*_*PVref*_) is the reference voltage, and *x*_1_ is the input PV voltage. When *e*_1_ is set to zero, we get the expected results. Taking the derivative of Eq (27) and substituting the dynamic model equation mentioned in Eq (13), we get:

$$\begin{array}{c} {\dot{e}}_{1}=\frac{i_{PV}}{C_{1}}-\frac{\mu x_{2}}{C_{1}}-{\dot{V}}_{PVref} \end{array}$$ (28)

The variable *x*_2_ behaves as a virtual control input in Eq (28). Subsequently, a Lyapunov function is selected. For all *x*, it must be radially unbounded and positive definite, and its time derivative must be negative definite for all *x* to ensure a local asymptotically stable solution [47]. The selected function and its time derivative along with Eq (28) are as follows:

$$\begin{array}{c} V_{1}=\frac{1}{2}e_{1}^{2} \end{array}$$ (29)

$$\begin{array}{c} {\dot{V}}_{1}=e_{1}{\dot{e}}_{1}=e_{1}(\frac{i_{PV}}{C_{1}}-\frac{\mu x_{2}}{C_{1}}-{\dot{V}}_{PVref}) \end{array}$$ (30)

To ensure ${\dot{V}}_{1}$ is negative, We substituted −*K*_1_*e*_1_ − *K*_2_sign(*e*_1_) in the parenthesis term in Eq (30). Now, *x*_2_ becomes:

$$\begin{array}{c} x_{2}=(K_{1}e_{1}+\frac{i_{PV}}{C_{1}}-\dot{V_{PVref}}+K_{2}\text{sign}\left(e_{1}\right))\frac{C_{1}}{\mu } \end{array}$$ (31)

Using the value of *x*_2_ from Eq (31), Eq (30) becomes,

$$\begin{array}{c} {\dot{V}}_{1}=-K_{1}e_{1}^{2}-K_{2}e_{1}\text{sign}\left(e_{1}\right) \end{array}$$ (32)

Eq (32) shows that for the Lyapunov function derivative to be negative definite, it is necessary that *K*_1_ and *K*_2_ must be positive, and Eq (31) must also be satisfied. Now, for *x*_2_, the inductor current needs a reference current denoted as *α*, serving as a stabilizing function. Additionally, the term λ*θ* is added for robustness, which represents the integral action term where *θ* is equal to the integral of *e*_1_.

$$\begin{array}{c} \alpha =(C_{1}K_{1}e_{1}+i_{PV}-C_{1}{\dot{V}}_{PVref}+K_{2}C_{1}\text{sign}\left(e_{1}\right))\frac{1}{\mu }+\lambda \theta \end{array}$$ (33)

Now, the reference current *α* follows the inductor current *x*_2_, and *e*_2_ must be zero.

$$\begin{array}{c} e_{2}=x_{2}-\alpha \end{array}$$ (34)

or,

$$\begin{array}{c} x_{2}=e_{2}+\alpha \end{array}$$ (35)

Putting Eq (35) in Eq (28), we get:

$$\begin{array}{c} {\dot{e}}_{1}=-K_{1}e_{1}-K_{2}\text{sign}\left(e_{1}\right)-\frac{\mu e_{2}}{C_{1}} \end{array}$$ (36)

Now, using Eq (36), Eq (30) becomes,

$$\begin{array}{c} {\dot{V}}_{1}=-K_{1}e_{1}^{2}-K_{2}e_{1}\text{sign}\left(e_{1}\right)-\frac{\mu e_{1}e_{2}}{C_{1}} \end{array}$$ (37)

Now, take the derivate of the second error, as defined in Eq (34),

$$\begin{array}{c} {\dot{e}}_{2}={\dot{x}}_{2}-\dot{\alpha } \end{array}$$ (38)

Also differentiate Eq (33) w.r.t time,

$$\begin{array}{c} \dot{\alpha }=\frac{1}{\mu^{2}}(\left(K_{1}{\dot{e}}_{1}C_{1}+{\dot{i}}_{PV}-{\ddot{V}}_{PVref}C_{1}\right)\mu -(K_{1}e_{1}C_{1}+\\ i_{PV}-C_{1}{\dot{V}}_{PVref}+K_{2}C_{1}\text{sign}\left(e_{1}\right))\dot{\mu })+\lambda e_{1} \end{array}$$ (39)

Putting Eq (36) in the above equation, we get:

$$\begin{array}{c} \dot{\alpha }=\frac{1}{\mu }(-K_{1}^{2}e_{1}C_{1}-K_{1}K_{2}C_{1}\text{sign}\left(e_{1}\right)+i_{PV}-{\ddot{V}}_{PVref}C_{1})\mu \\ -\frac{\alpha \dot{\mu }}{\mu }-K_{1}e_{2}+\lambda e_{1} \end{array}$$ (40)

Substituting Eqs (14) and (40) in Eq (38), we get:

$$\begin{array}{c} {\dot{e}}_{2}=-\frac{x_{3}}{L}+\mu \frac{x_{1}+x_{3}}{L}-\frac{1}{\mu }(-K_{1}^{2}e_{1}C_{1}-K_{1}K_{2}C_{1}\text{sign}\left(e_{1}\right)\\ +i_{PV}-{\ddot{V}}_{PVref}C_{1})+\frac{\alpha \dot{\mu }}{\mu }+K_{1}e_{2}-\lambda e_{1} \end{array}$$ (41)

Now, to ensure system asymptotic stability and the convergence of error *e*_1_ and error *e*_2_ to zero, we check the Lyapunov composite function for this system.

$$\begin{array}{c} V_{2}=V_{1}+\frac{1}{2}e_{2}^{2} \end{array}$$ (42)

The time derivative of the above equation along with Eq (37) is,

$$\begin{array}{c} {\dot{V}}_{2}={\dot{V}}_{1}+e_{2}{\dot{e}}_{2}=-K_{1}e_{1}^{2}-K_{2}e_{1}\text{sign}\left(e_{1}\right)+e_{2}({\dot{e}}_{2}-\mu \frac{e_{1}}{C_{1}}) \end{array}$$ (43)

For the Lyapunov function derivative to be negative we substitute parenthesis term of Eq (37) to −*K*_3_*e*_2_ − *K*_4_sign(*e*_2_), and we get,

$$\begin{array}{c} {\dot{e}}_{2}-\mu \frac{e_{1}}{C_{1}}=-K_{3}e_{2}-K_{4}\text{sign}\left(e_{2}\right) \end{array}$$ (44)

*K*_3_ and *K*_4_ must be positive definite. Put Eq (41) in Eq (44),

$$\begin{array}{c} -K_{3}e_{2}-K_{4}\text{sign}\left(e_{2}\right)=-\frac{x_{3}}{L}+\mu \frac{x_{1}+x_{3}}{L}-\frac{1}{\mu }(-K_{1}^{2}e_{1}C_{1}-\\ K_{1}K_{2}C_{1}\text{sign}\left(e_{1}\right)+i_{PV}-{\ddot{V}}_{PVref}C_{1})+\frac{\alpha \dot{\mu }}{\mu }+K_{1}e_{2}-\lambda e_{1}\\ -\mu \frac{e_{1}}{C_{1}} \end{array}$$ (45)

Solving for $\dot{\mu }$ the final expression for control law is shown in Eq (46),

$$\begin{array}{c} \dot{\mu }=\frac{1}{\alpha }[e_{2}\left(-K_{1}-K_{3}\right)\mu -e_{1}(C_{1}K_{1}^{2}-\frac{\mu^{2}}{C_{1}}-\mu \lambda )+\mu \frac{x_{3}}{L}]+\\ \frac{1}{\alpha }[{\dot{i}}_{PV}-C_{1}{\ddot{V}}_{PVref}-\mu^{2}(\frac{x_{1}+x_{3}}{L})-K_{1}K_{2}C_{1}\text{sign}\left(e_{1}\right)]+\\ \frac{\mu }{\alpha }[-K_{4}\text{sign}\left(e_{2}\right)] \end{array}$$ (46)

Where *α* ≠ 0 and 0 < *μ* < 1.

Eq (46) shows the control law for a buck-boost converter using a robust integral back-stepping controller.

### 3.2 Control law for inverter using robust integral back-stepping controller

The objective is to use a Robust Integral Back-Stepping Controller (RIBSC) to generate a sinusoidal output voltage at the load terminal, facilitating closed-loop control. The RIBSC structure is primarily based on the Lyapunov dynamic system stability theory [48]. This process is intended to force the inverter output voltage *x*_1_ closely track reference voltage *U*_*cref*_ with the highest degree of robustness and minimal THD. Therefore, *e*_3_ represents the error, defined in Eq (47).

$$\begin{array}{c} e_{3}=U_{cref}-x_{1} \end{array}$$ (47)

The goal is to get *e*_3_ close to zero. Taking the derivative of Eq (47) and substituting the dynamic model equation described in Eq (23), we get:

$$\begin{array}{c} {\dot{e}}_{3}={\dot{U}}_{cref}-\frac{x_{2}}{C}+\frac{i_{o}}{C} \end{array}$$ (48)

Choosing a Lyapunov function, and the time derivative of the Lyapunov function along with Eq (48) is as follows:

$$\begin{array}{c} V_{1}=\frac{1}{2}e_{3}^{2} \end{array}$$ (49)

$$\begin{array}{c} {\dot{V}}_{1}=e_{3}{\dot{e}}_{3}=e_{3}({\dot{U}}_{cref}-\frac{x_{2}}{C}+\frac{i_{o}}{C}) \end{array}$$ (50)

Second error is defined as follows,

$$\begin{array}{c} e_{4}=\alpha -x_{2} \end{array}$$ (51)

or,

$$\begin{array}{c} x_{2}=\alpha -e_{4} \end{array}$$ (52)

So, the derivative of Eq (51) is,

$$\begin{array}{c} {\dot{e}}_{4}=\dot{\alpha }-{\dot{x}}_{2} \end{array}$$ (53)

Putting Eq (24) in Eq (53), we get:

$$\begin{array}{c} {\dot{e}}_{4}=\dot{\alpha }+\frac{x_{1}}{L_{F}}-\frac{\mu_{2}V_{c2}}{L_{F}} \end{array}$$ (54)

From Eq (50), substituting −*K*_3_*e*_3_ − *K*_4_sign(*e*_3_) for robustness,

$$\begin{array}{c} x_{2}=C{\dot{U}}_{cref}+K_{3}e_{3}C+i_{o}+CK_{4}\text{sign}\left(e_{3}\right) \end{array}$$ (55)

For *V*_1_ to be negative definite, *K*_3_ and *K*_4_ must be positive definite, and Eq (55) must satisfy *α*. Additionally, the term λ*θ* is added for robustness, representing the integral action term where *θ* is equal to the integral of *e*_3_. So,

$$\begin{array}{c} \alpha =(C{\dot{U}}_{cref}+K_{3}e_{3}C+i_{o}+CK_{4}\text{sign}\left(e_{3}\right))+\lambda \theta \end{array}$$ (56)

Putting Eq (52) in Eq (50), we get:

$$\begin{array}{c} {\dot{V}}_{1}=e_{3}({\dot{U}}_{cref}-\frac{\alpha }{C}+\frac{i_{o}}{C}+\frac{e_{4}}{C}) \end{array}$$ (57)

Putting the value of *α* into the above equation, we get:

$$\begin{array}{c} {\dot{V}}_{1}=-K_{3}e_{3}^{2}+\frac{e_{3}e_{4}}{C}-K_{4}e_{3}\text{sign}\left(e_{3}\right) \end{array}$$ (58)

Taking the derivative of Eq (56) and its simplified form is as follows,

$$\begin{array}{c} \dot{\alpha }=C{\ddot{U}}_{cref}+K_{3}{\dot{e}}_{3}C+i_{o}+0+\lambda e_{3} \end{array}$$ (59)

$$\begin{array}{c} \dot{\alpha }=C{\ddot{U}}_{cref}-K_{3}^{2}e_{3}C-CK_{4}K_{3}\text{sign}\left(e_{3}\right)+i_{o}+\lambda e_{3} \end{array}$$ (60)

Choosing a second Lyapunov function,

$$\begin{array}{c} V_{2}=V_{1}+\frac{1}{2}e_{4}^{2} \end{array}$$ (61)

Its derivative along Eq (58) is,

$$\begin{array}{c} {\dot{V}}_{2}={\dot{V}}_{1}+e_{4}{\dot{e}}_{4}=-K_{3}e_{3}^{2}-K_{4}e_{3}\text{sign}\left(e_{3}\right)+e_{4}(\frac{e_{3}}{C}+{\dot{e}}_{4}) \end{array}$$ (62)

Putting Eq (54) in Eq (62), we get:

$$\begin{array}{c} {\dot{V}}_{2}=-K_{3}e_{3}^{2}-K_{4}e_{3}\text{sign}\left(e_{3}\right)+e_{4}(\frac{e_{3}}{C}+\dot{\alpha }-\frac{\mu_{2}V_{c2}}{L_{F}}+\frac{x_{1}}{L_{F}}) \end{array}$$ (63)

Putting Eq (60) in Eq (63), we get:

$$\begin{array}{c} {\dot{V}}_{2}=-K_{3}e_{3}^{2}-\frac{K_{4}e_{3}\text{sign}\left(e_{3}\right)}{C}+e_{4}(\frac{e_{3}}{C}+C{\ddot{U}}_{cref}-K_{3}^{2}e_{3}C-\\ CK_{4}K_{3}\text{sign}\left(e_{3}\right)+i_{o}+\lambda e_{3}-\frac{\mu_{2}V_{c2}}{L_{F}}+\frac{x_{1}}{L_{F}}) \end{array}$$ (64)

$$\begin{array}{c} \frac{e_{3}}{C}+C{\ddot{U}}_{cref}-K_{3}^{2}e_{3}C-CK_{4}K_{3}\text{sign}\left(e_{3}\right)+i_{o}+\lambda e_{3}-\frac{\mu_{2}V_{c2}}{L_{F}}\\ +\frac{x_{1}}{L_{F}}=-K_{5}e_{4}-K_{6}\text{sign}\left(e_{4}\right) \end{array}$$ (65)

To get ${\dot{V}}_{2}<0$ a control law *μ*_2_ is chosen from the inverter defined in Eq (66),

$$\begin{array}{c} \mu_{2}=\frac{L_{F}}{V_{c2}}(\frac{e_{3}}{C}+C{\ddot{U}}_{cref}-K_{3}^{2}e_{3}C-CK_{4}K_{3}\text{sign}\left(e_{3}\right)+i_{o}+\\ \lambda e_{3}+\frac{x_{1}}{L_{F}}+K_{5}e_{4}+K_{6}\text{sign}\left(e_{4}\right)) \end{array}$$ (66)

The error *e*_3_ tends to zero with the application of this control rule to the PWM inverter in the standalone mode because the derivative of *V*_1_ and *V*_2_ is negative.

## 4. Simulation results

To analyze the overall performance of the PV system with the inverter using the proposed technique, MATLAB/Simulink is used. The system parameters are listed in Table 1, and the controller parameters can be found in Table 2. The proposed technique is evaluated in two different scenarios: 1) Steady-state performance of the inverter output voltage with different loads 2) Transient performance of the inverter output voltage with different loads when the reference amplitude changes in steps. The performance of the proposed technique is analyzed and compared with the back-stepping and PID controllers.

**Table 1:** System parameters.

Types	Parameter	Symbol	Value	Unit
**PV module**	Maximum power	*P*_*max*_	1555	W
	Number of cells per module	*N*_*s*_	72	
	Voltage at maximum power	*V*_*mp*_	102.6	V
	Open circuit voltage	*V*_*oc*_	165.8	V
	Short circuit current	*I*_*sc*_	17.56	A
	Current at maximum power	*I*_*mp*_	15.16	A
**Converter**	Converter input capacitor	*C*_1_	1	mF
	Converter output capacitor	*C*_2_	48	μF
	Converter inductor	L	20	mH
	Switching frequency	*f*_*s*_	5	kHz
**Inverter and LC filter**	Inductor	*L*_*F*_	47	mH
	capacitor	C	150	μF
	Switching frequency	*f*_*s*_	20	kHz

**Table 2:** Controllers parameters.

Type	Controllers Constant Parameter	Symbol	Value
Converter RIBSC	Constant	*K*_1_	100
	Constant	*K*_2_	9000
	Constant	*K*_3_	2000
	Constant	*K*_4_	10
	Constant	λ	29
Inverter RIBSC	Constant	*K*_3_	70000
	Constant	*K*_4_	20000
	Constant	*K*_5_	70000
	Constant	*K*_6_	20000
	Constant	λ	100

### 4.1 Steady-state performance of the inverter output voltage with different loads

**Fig 10:**
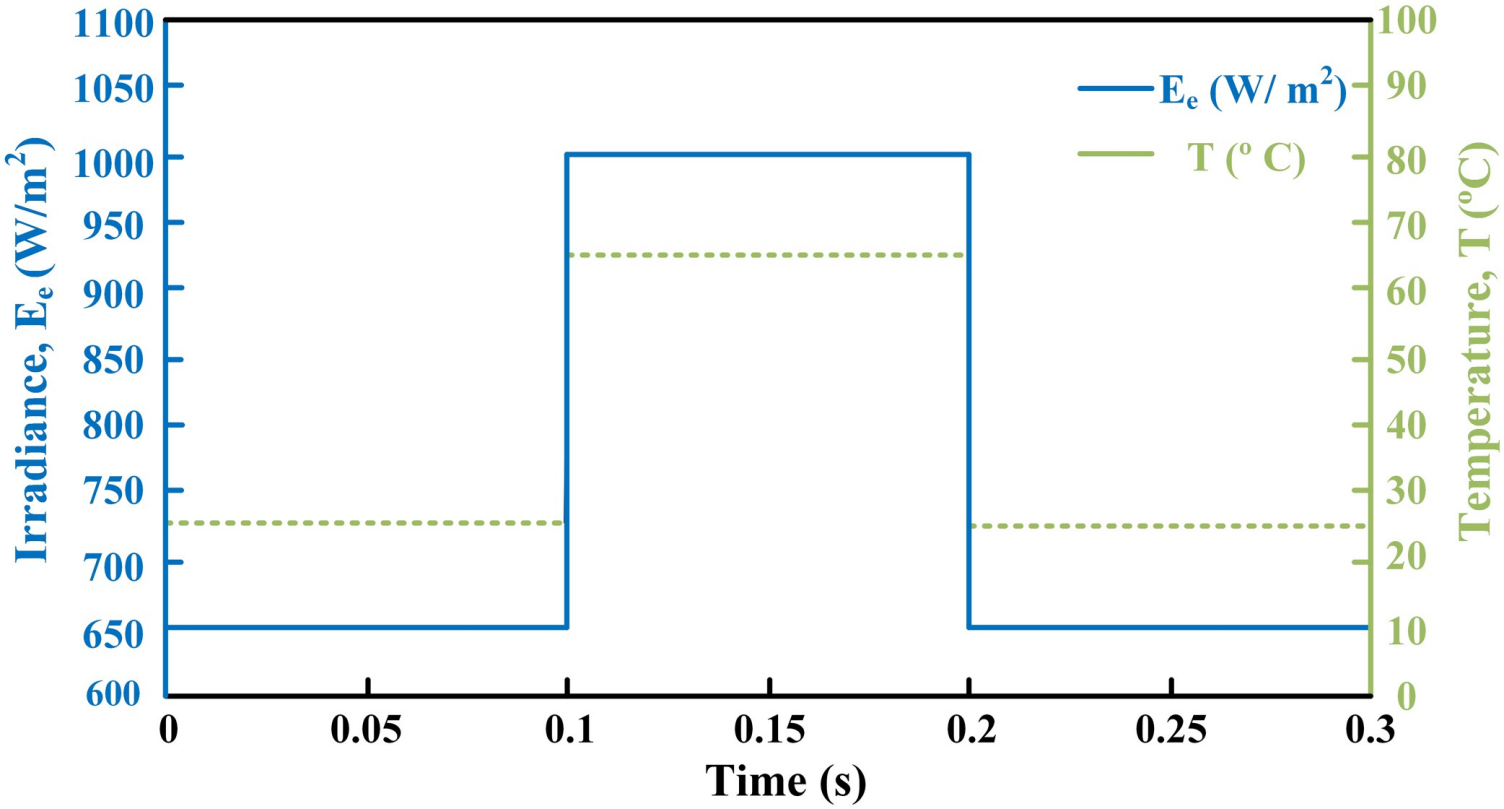
Varying temperature and irradiance profile.

**Fig 11:**
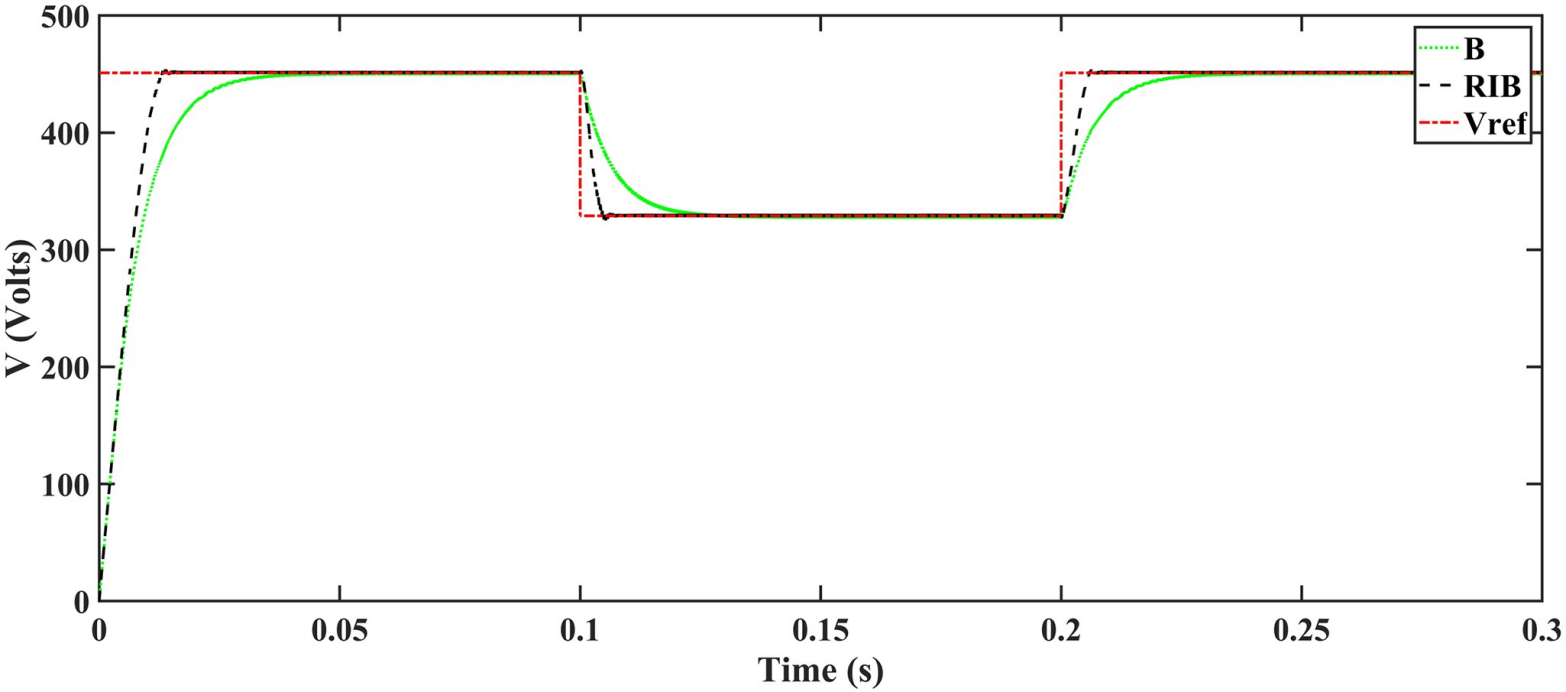
Output voltage of the PV array.

**Fig 12:**
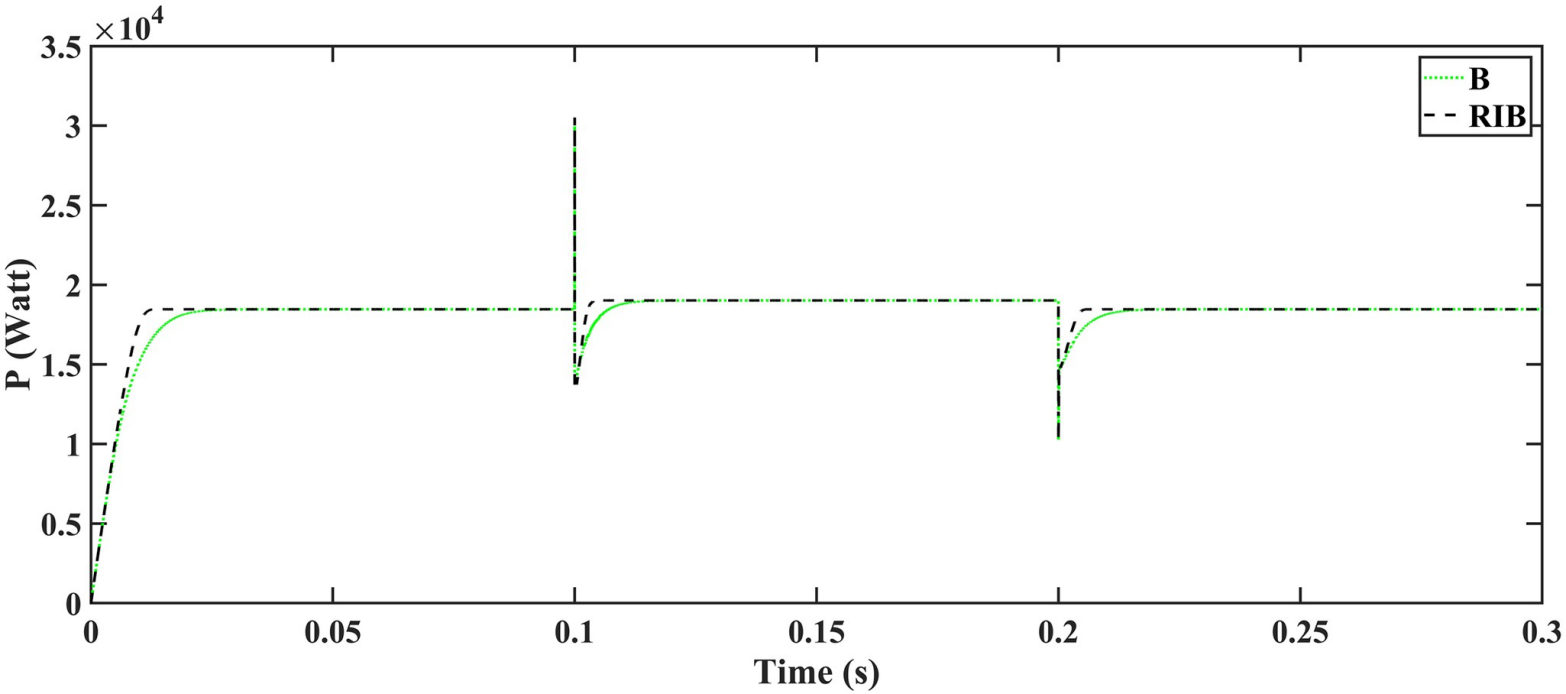
Output power of the PV array.

**Fig 13:**
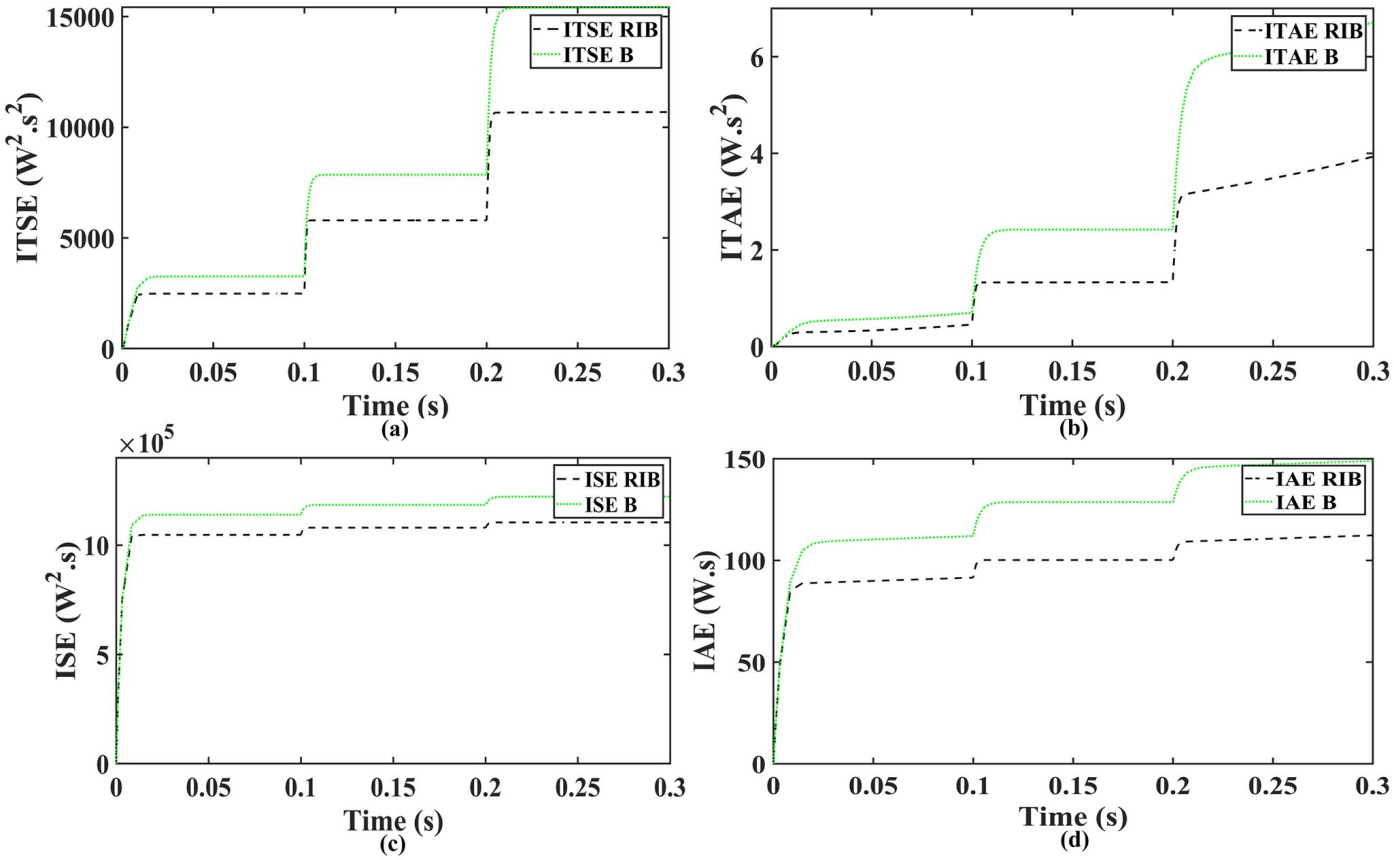
Performance indices of PV array output power (a)ITSE (b)ITAE (c)ISE (d)IAE.

Fig 10 illustrates the profile of varying temperature and irradiance. The temperature is initially held at 25ºC, and the irradiance at 650*W*/*m*^2^ from 0s to 0.1s. Between 0.1s and 0.2s, the temperature changes to 65ºC, and the irradiance to 1000*W*/*m*^2^. In the subsequent time interval, the temperature is reverted to 25ºC, and the irradiance to 650*W*/*m*^2^. The reference voltage, generated through the regression plane, is successfully tracked by the PV array output voltage, as shown in Fig 11. It is evident from the results that the RIBSC controller reaches a steady-state condition in less than 0.15s compared to other benchmark controllers. PV module output power under fluctuating environmental conditions are shown in Fig 12. The output power is 18.4kW from 0.1s to 0.2s, 19.02kW for the second interval, and the same as the first interval for the last interval. The performance criteria (i.e. ITSE, ITAE, ISE, and IAE, where the error here is the difference between the reference power and the output power of PV array) assesses the robustness of the proposed controller. Fig 13 shows that the proposed controller accumulates less error compared to the back-stepping controller. The formulas for calculating these errors are as follows:

$$\begin{array}{c} ITSE=\int_{t_{0}}^{t_{f}}te{\left(t\right)}^{2}dt \end{array}$$ (67)

$$\begin{array}{c} ITAE=\int_{t_{0}}^{t_{f}}t\left|e\left(t\right)\right|dt \end{array}$$ (68)

$$\begin{array}{c} ISE=\int_{t_{0}}^{t_{f}}e{\left(t\right)}^{2}dt \end{array}$$ (69)

$$\begin{array}{c} IAE=\int_{t_{0}}^{t_{f}}\left|e\left(t\right)\right|dt \end{array}$$ (70)

**Fig 14:**
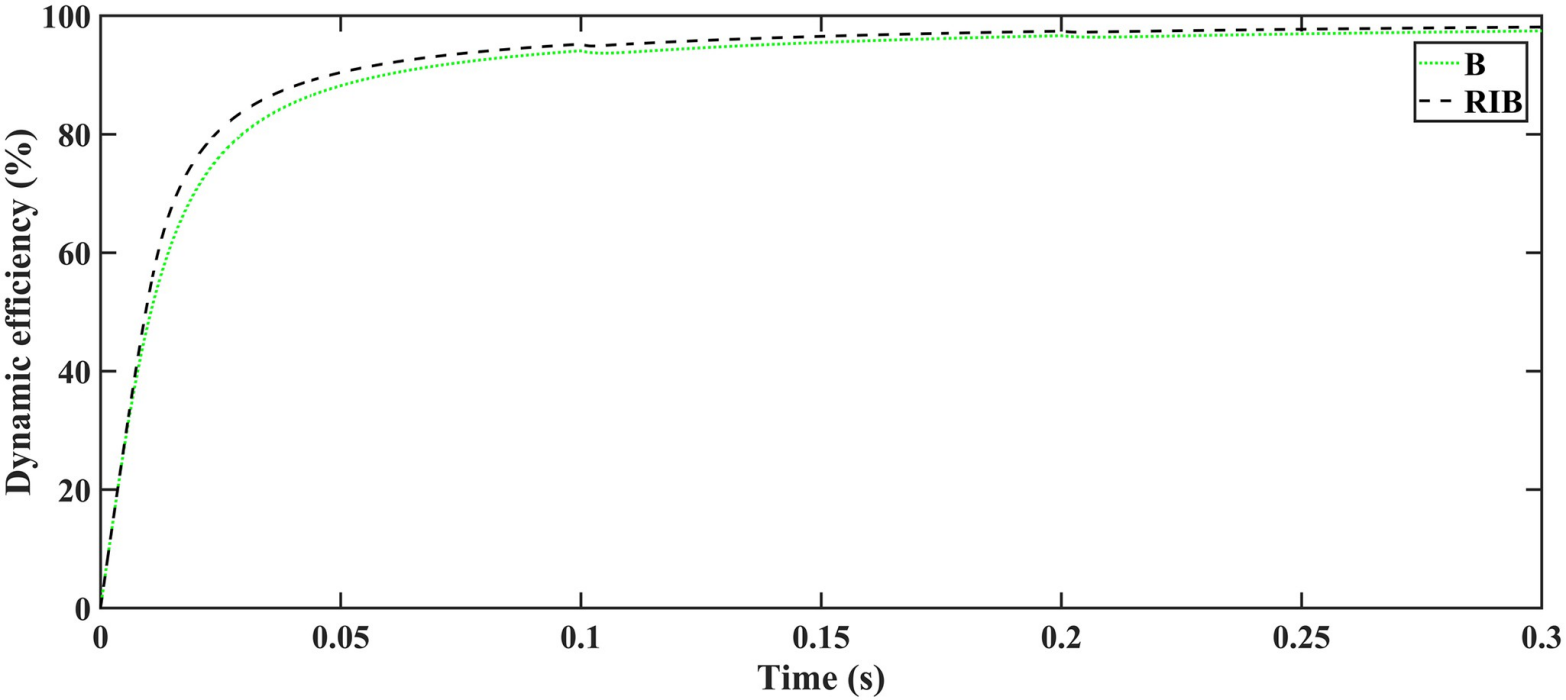
Dynamic efficiency.

The dynamic efficiency of back-stepping is 97.45%, while for robust integral back-stepping, it is 98.11% at the MPPT stage, as shown in Fig 14. The efficiency of MPPT controller is compared with other state-of-the-art techniques in Table 3. Dynamic efficiency is defined as the ratio of PV output power to the PV power at MPP over a specific time period, and it is defined as;

$$\eta_{100}=\frac{\int_{t_{0}}^{t_{f}}P_{PV}dt}{\int_{t_{0}}^{t_{f}}P_{MPP}dt}\times 100=\frac{\int_{t_{0}}^{t_{f}}\left(V_{PV}\times I_{PV}\right)dt}{\int_{t_{0}}^{t_{f}}P_{MPP}dt}\times 100$$ (71)

**Table 3:** MPPT dynamic efficiency.

Algorithms	Efficiency (%)
P & O	96
PI	96.5
Back-stepping	97.45
Robust integral back-stepping (Proposed)	98.11

Because of the robustness, we use robust integral back-stepping for all scenarios. The MPPT is unaffected and remains the same for all loads.

#### 4.1.1 Scenario 1

**Fig 15:**
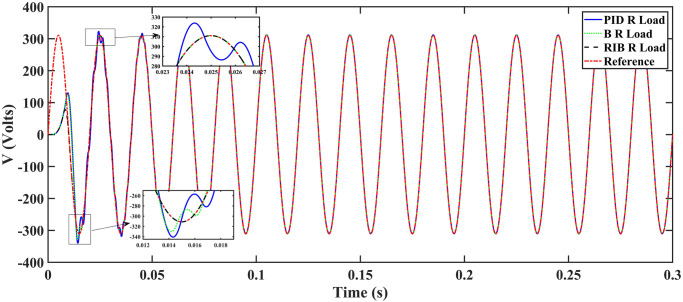
Inverter output voltage at resistive load after filtering.

In scenario 1, a resistive load is considered, and the inverter’s performance with different controllers are depicted in Fig 15. The plot depicts four wave-forms: the inverter’s output voltage at the load terminal *V*_*C*_ for three controllers and the sinusoidal reference signal *U*_*cref*_. The results show that the output voltage perfectly tracks the reference voltage, which is a sinusoidal waveform. The proposed controller tracks the reference voltage in almost 0.01s, while the back-stepping and PID controllers do so in 0.025s and 0.04s, respectively. As can be seen from Fig 15, the initial response of PID and back-stepping controllers have overshoots and undershoots, whereas the proposed controller has readily tracked the reference voltage from the start without any overshoot and undershoot. This also implies the convergence time of the proposed controller is smaller. When the inverter’s input voltage is below 700V which is the nominal input voltage of the inverter, a drop appears in the output voltage. The distortion in the output voltage at the beginning is due to the insufficient voltage provided by the non-inverting buck-boost converter. After 0.01s, in the case of the proposed controller, the input voltage of the inverter exceeds 700V and the output voltage of the inverter begins to track the reference voltage. The frequency analysis of the output voltage and Root Means Square Error (RMSE) are compared in Tables 4 and 5, respectively. RMSE is a commonly used measure for evaluating the quality of actual values, indicating how far the actual values deviate from the true measured values. To compute RMSE, the residual (the difference between prediction and truth) for each data point is calculated, norm of residuals for each data point are found, mean of residuals is computed, and then square root of the resulting mean is computed.

$$RMSE=\sqrt{\frac{\Sigma_{i=1}^{N}|V_{ref}-V_{out}{|}^{2}}{N}}$$ (72)

**Table 4:** Comparison of THD of different control techniques.

Scenario	Normal Operation			Step changes in reference amplitude		
Control Techniques	Scenario 1 (%)	Scenario 2 (%)	Scenario 3 (%)	Scenario 4 (%)	Scenario 5 (%)	Scenario 6 (%)
**PID**	13.18	13.18	20.13	14.27	14.28	21.57
**Back-stepping**	12.66	12.66	14.22	13.82	13.82	15.76
**Robust Integral Back-stepping**	9.71	9.72	13.04	11.45	11.45	14.33

**Table 5:** Root Means Square Error (RMSE) of reference voltage and output voltage of inverter.

Scenario	Normal Operation			Step changes in reference amplitude		
Control Techniques	Scenario 1	Scenario 2	Scenario 3	Scenario 4	Scenario 5	Scenario 6
**PID**	0.4523	0.4524	0.833	0.4529	0.4536	0.888
**Back-stepping**	0.4365	0.4365	0.586	0.4371	0.4371	0.6025
**Robust Integral Back-stepping**	0.3998	0.40	0.5477	0.408	0.409	0.5624

The fundamental voltage is about 300.8V, 301.3V, and 301.8V for the PID controller, back-stepping controller and proposed controller, respectively, at a frequency of 50Hz. The THD for these controllers is 13.18%, 12.66%, and 9.71%. THD measures the total amount of harmonic distortion present in a current signal and is expressed as a percentage, as follows

$$THD\%=\frac{\sqrt{\Sigma_{n=1}^{n=max}I_{n}^{2}}}{I_{0}}$$ (73)

**Fig 16:**
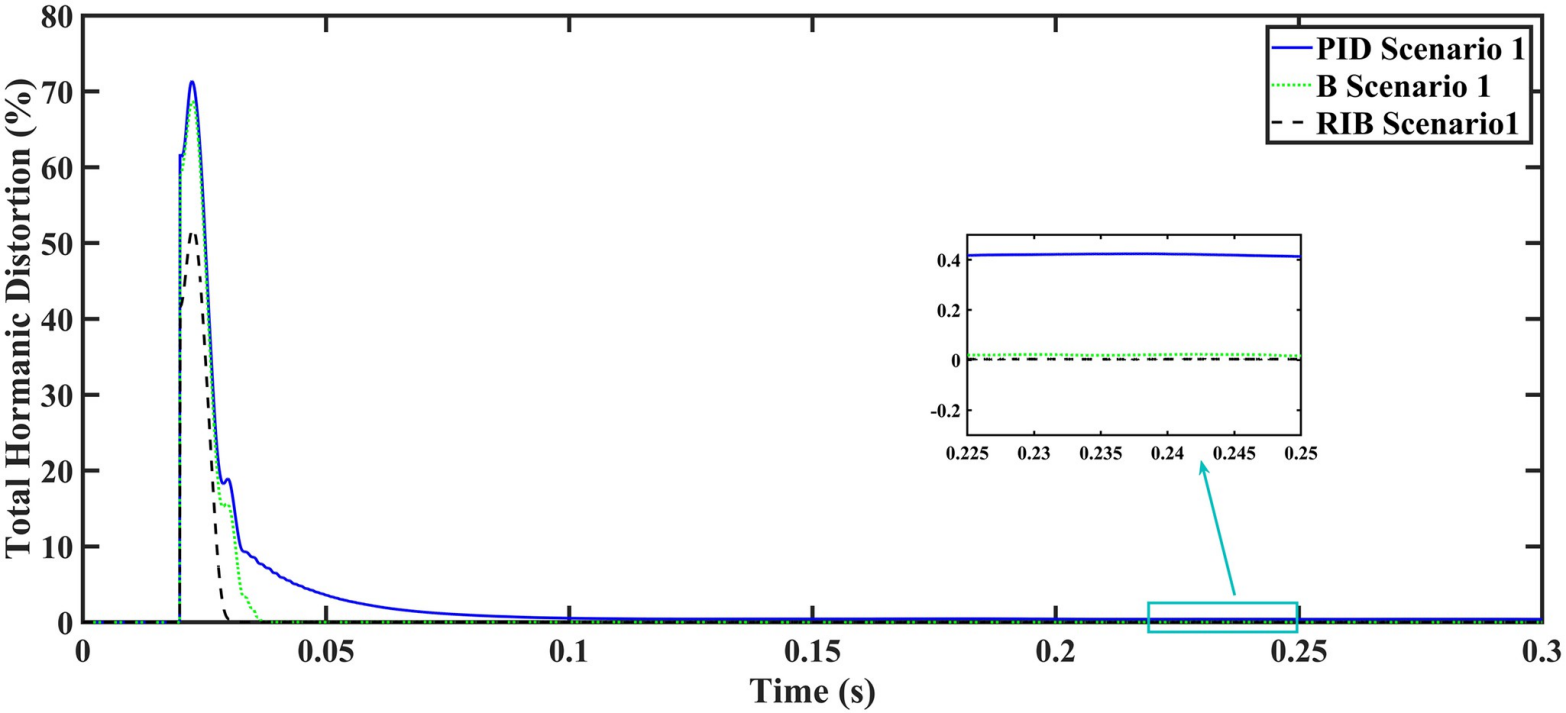
Total harmonic distortion.

**Fig 17:**
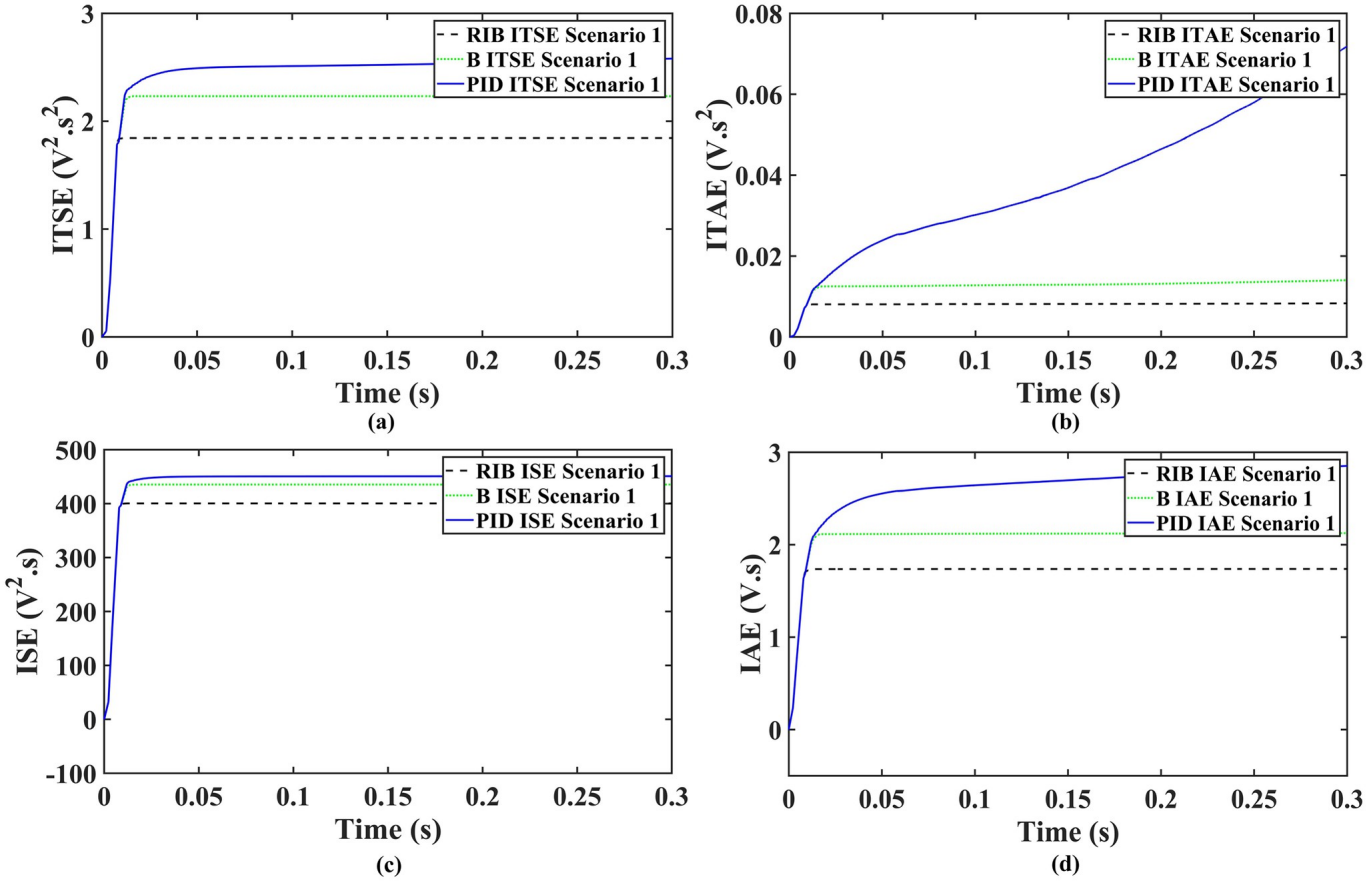
Performance indices of inverter output voltage with resistive load (a)ITSE (b)ITAE (c)ISE (d)IAE.

In this equation, *I*_0_ represents the fundamental component of the signal, and *I*_*n*_ stands for the nth order harmonics of the same signal. The RIB controller exhibits a lower harmonic distortion compared to the benchmark controllers, which is less than 5% according to the IEEE standard. As is evident from Fig 16, the THD due to proposed controller converges to zero level earlier than the PID and back-stepping controllers. In other words, the proposed controller as fewer harmonics in the output of the inverter. The performance indices of the inverter output voltage is calculated in Fig 17 and are compared in Table 6. Fig 17 shows the tracking error in terms of four error metrics. As can be seen from the figures, PID controller has the largest error and the proposed controller has the minimum error. In addition, Fig 17(b) and 17(d) shows that the tracking error due to PID controller keeps on increasing and does not converge in finite time. The inverter, evaluated with the suggested controller in standalone mode, displays low harmonic distortion, strong control performance, and a high-quality sinusoidal waveform, as can be concluded from Figs 15–17.

**Table 6:** Comparison of ITSE, ITAE, ISE and IAE under normal operation.

Scenario	Scenario 1			Scenario 2			Scenario 3		
Control Techniques	PID	B	RIB	PID	B	RIB	PID	B	RIB
**ITSE**	2.581	2.232	1.844	2.594	2.242	1.855	38.12	4.622	3.962
**ITAE**	0.0718	0.0140	0.0081	0.0720	0.0139	0.0083	0.991	0.0992	0.0864
**ISE**	450.8	435.3	400.3	451.6	436	401	834.8	587.6	548.4
**IAE**	2.852	2.124	1.738	2.865	2.121	1.742	9.423	3.339	3.181

#### 4.1.2 Scenario 2

**Fig 18:**
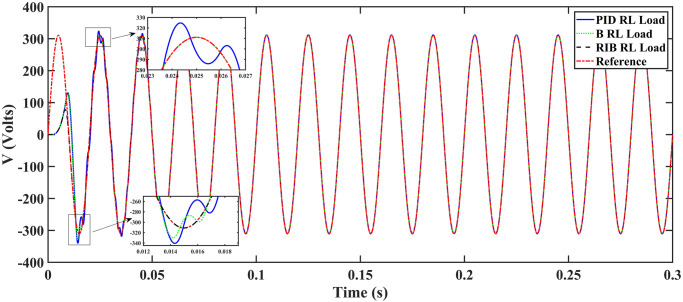
Inverter output voltage at resistive inductive loads after flirting.

**Fig 19:**
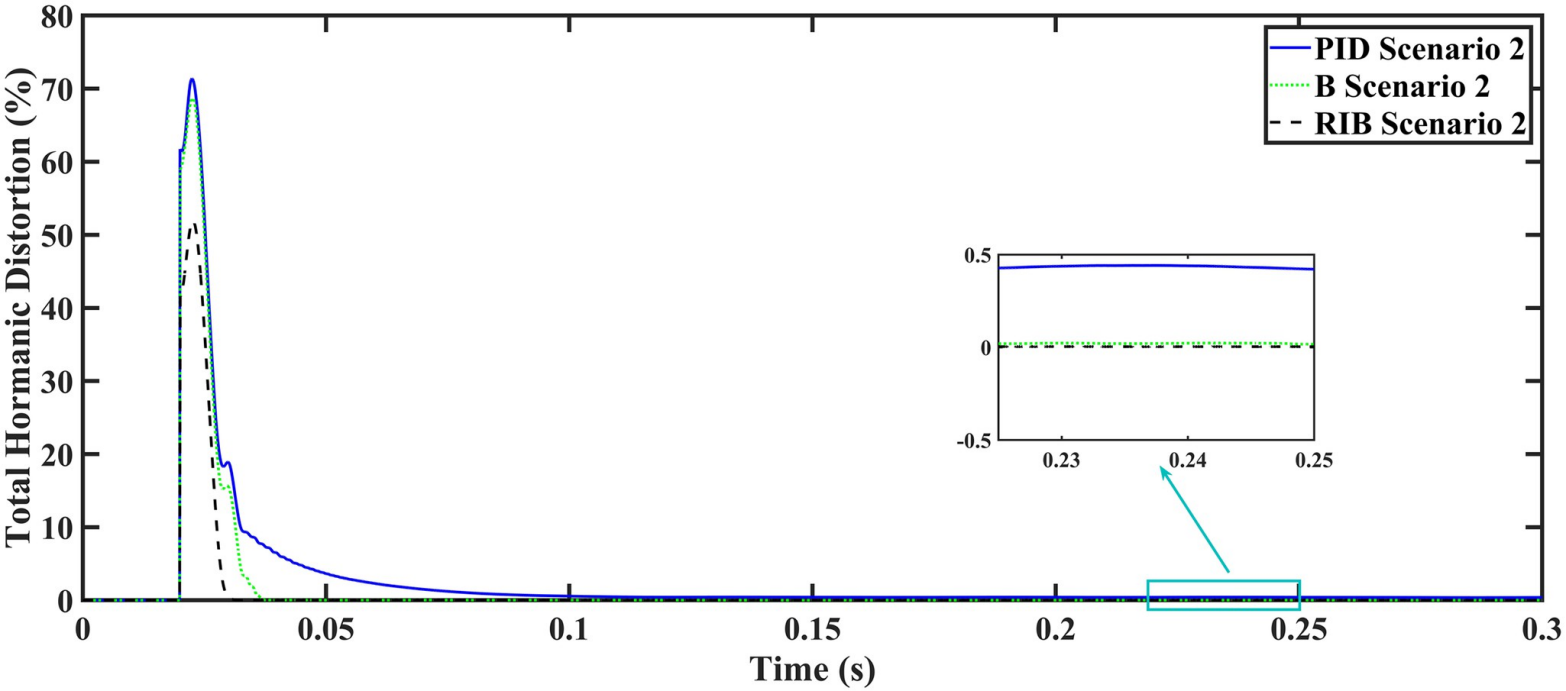
Total harmonic distortion.

**Fig 20:**
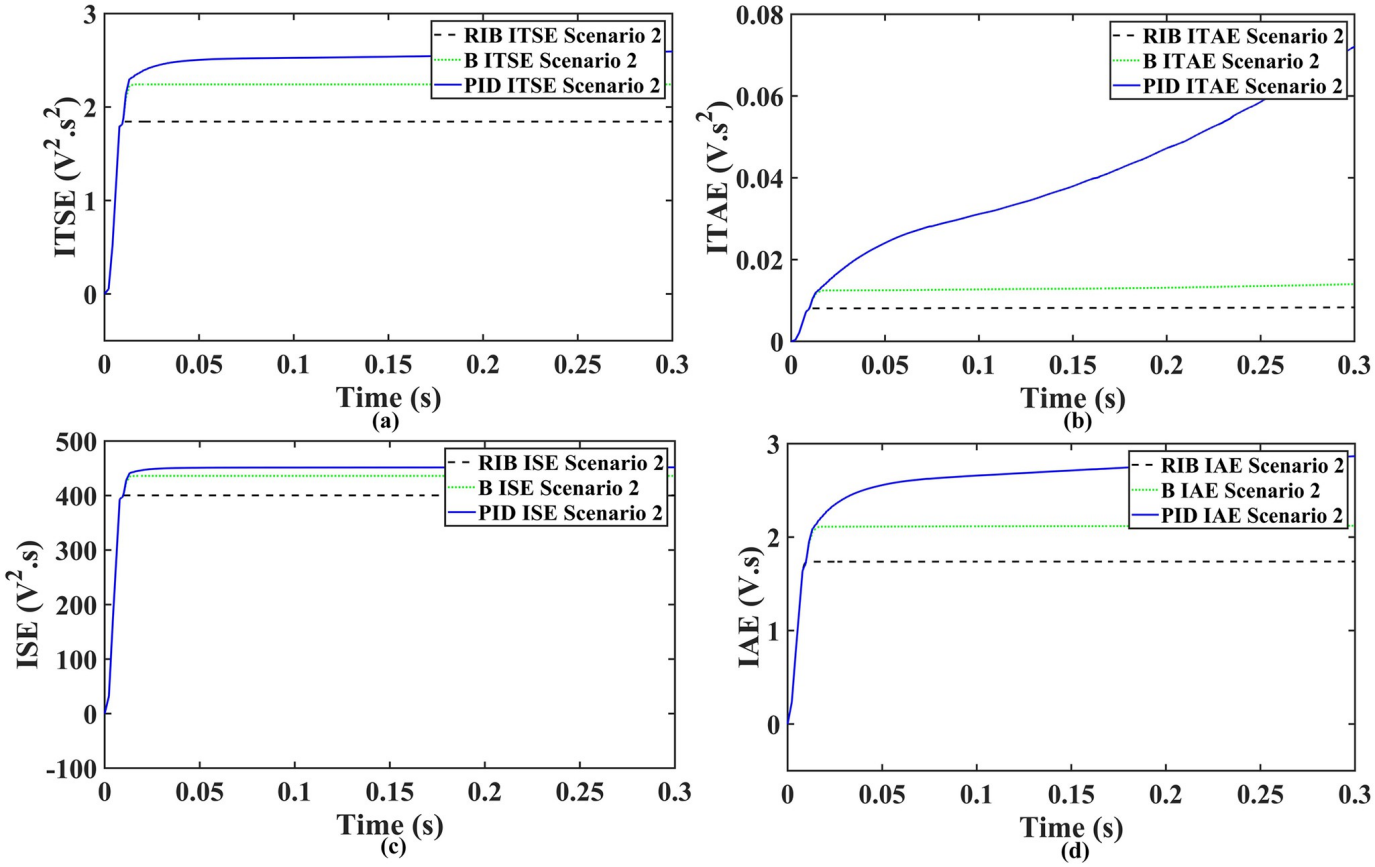
Performance indices of inverter output voltage with resistive inductive loads (a)ITSE (b)ITAE (c)ISE (d)IAE.

We assume resistive inductive loads in scenario 2. The performance of the inverter with different controllers are shown in Fig 18, depicting four wave-forms: the inverter’s output voltage at the terminal load *V*_*C*_ for the three controllers and the sinusoidal reference signal *U*_*cref*_. The results show that the output voltage perfectly tracks the reference voltage, which is a sinusoidal waveform. The proposed controller tracks the reference voltage in almost 0.01s, while the back-stepping controller and the PID controller track in 0.025s and 0.04s, respectively. As can be seen from Fig 18, the initial response of PID and back-stepping controllers have overshoots and undershoots, whereas the proposed controller has readily tracked the reference voltage from the start without any overshoot and undershoot. This also implies the convergence time of the proposed controller is smaller. If the inverter input voltage is less than 700V which is the nominal voltage of the inverter input, then a drop appears in the output voltage. The distortion in the output voltage of the inverter at the beginning is due to the insufficient voltage provided by the non-inverting buck-boost converter. After 0.01s, in the case of the proposed controller, the input voltage of the inverter exceeds 700V and the output voltage of the inverter begins to track the reference voltage. The output voltage frequency analysis and RMSE are compared in Tables 4 and 5, respectively. The fundamental voltage is about 300.8V, 301.3V, and 301.8V in the case of the PID controller, back-stepping controller, and proposed controller, respectively, at a 50Hz frequency, while the THD is 13.18%, 12.66%, and 9.72%. This means that the RMS magnitude of harmonic frequencies are 13.18%, 12.66%, and 9.72% of the RMS magnitude of the fundamental frequency. The RIB controller shows better harmonic distortion compared to the benchmark controllers, which is less than 5% according to the IEEE standard. As is evident from Fig 19, the THD due to proposed controller converges to zero level earlier than the PID and back-stepping controllers. In other words, the proposed controller as fewer harmonics in the output of the inverter. The performance indices of the inverter output voltage is calculated in Fig 20 and are compared in Table 6. Fig 20 shows the tracking error in terms of four error metrics. As can be seen from the figures, PID controller has the largest error and the proposed controller has the minimum error. In addition, Fig 17(b) and 17(d) shows that the tracking error due to PID controller keeps on increasing and does not converge in finite time. The inverter, evaluated with the suggested controller in standalone mode, exhibits low harmonic distortion, strong control performance, and a high-quality sinusoidal waveform, as can be concluded from Figs 18–20.

#### 4.1.3 Scenario 3

**Fig 21:**
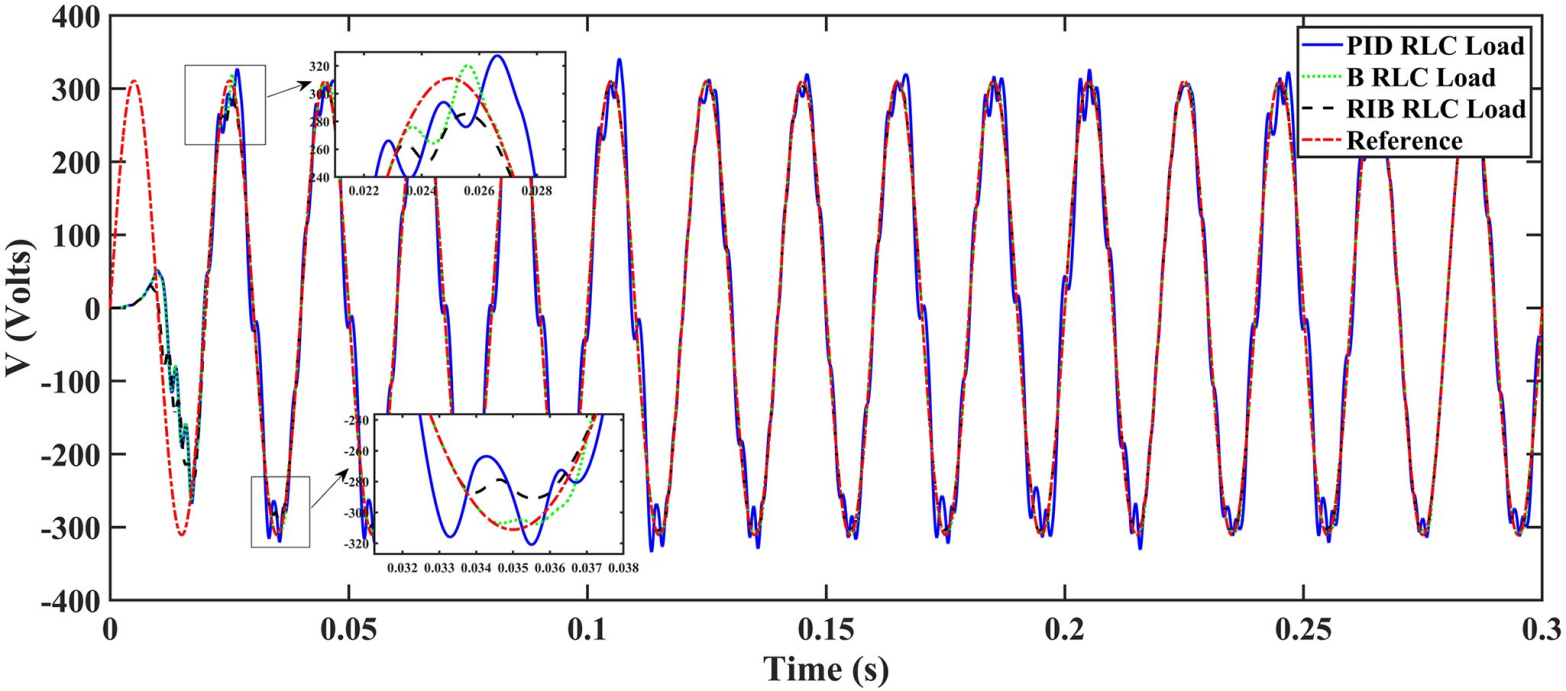
Inverter output voltage at resistive, inductive, and capacitive loads after filtering.

**Fig 22:**
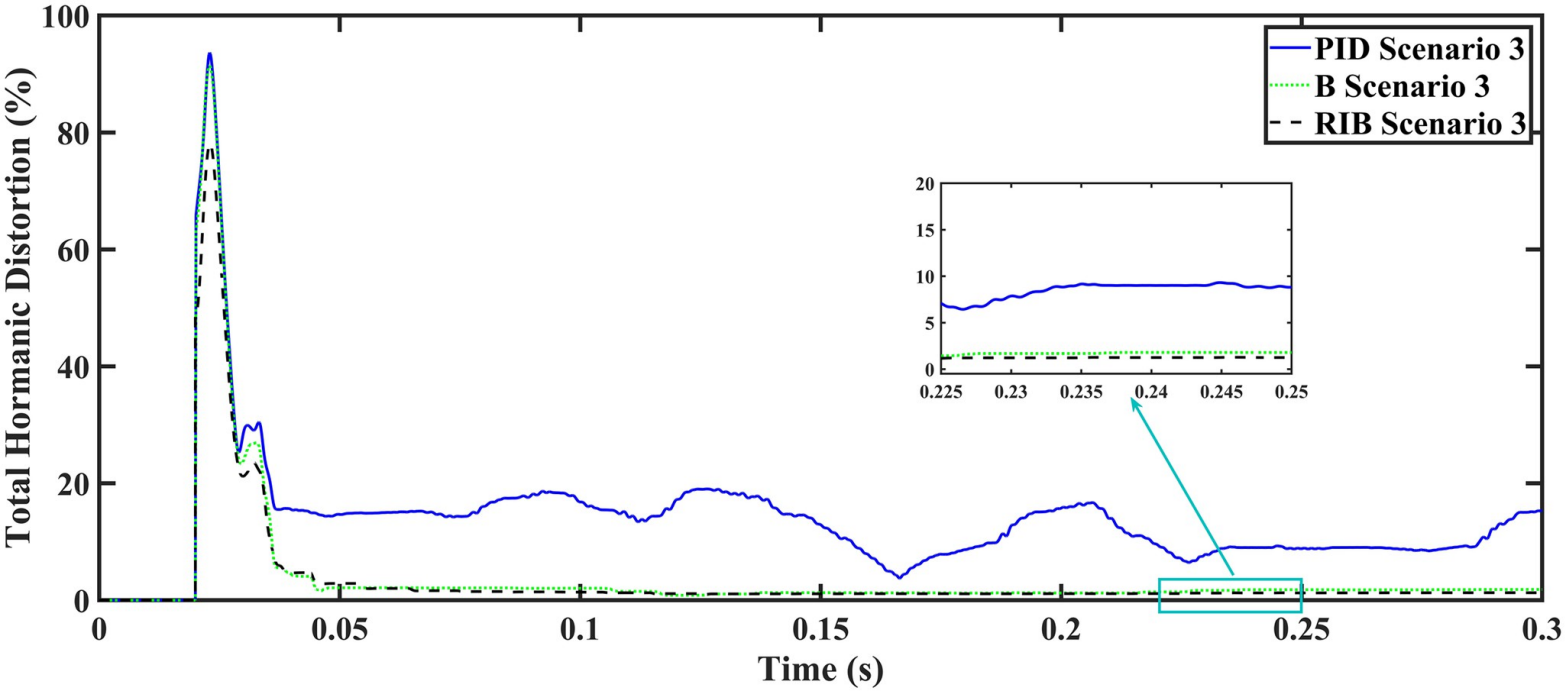
Total harmonic distortion.

**Fig 23:**
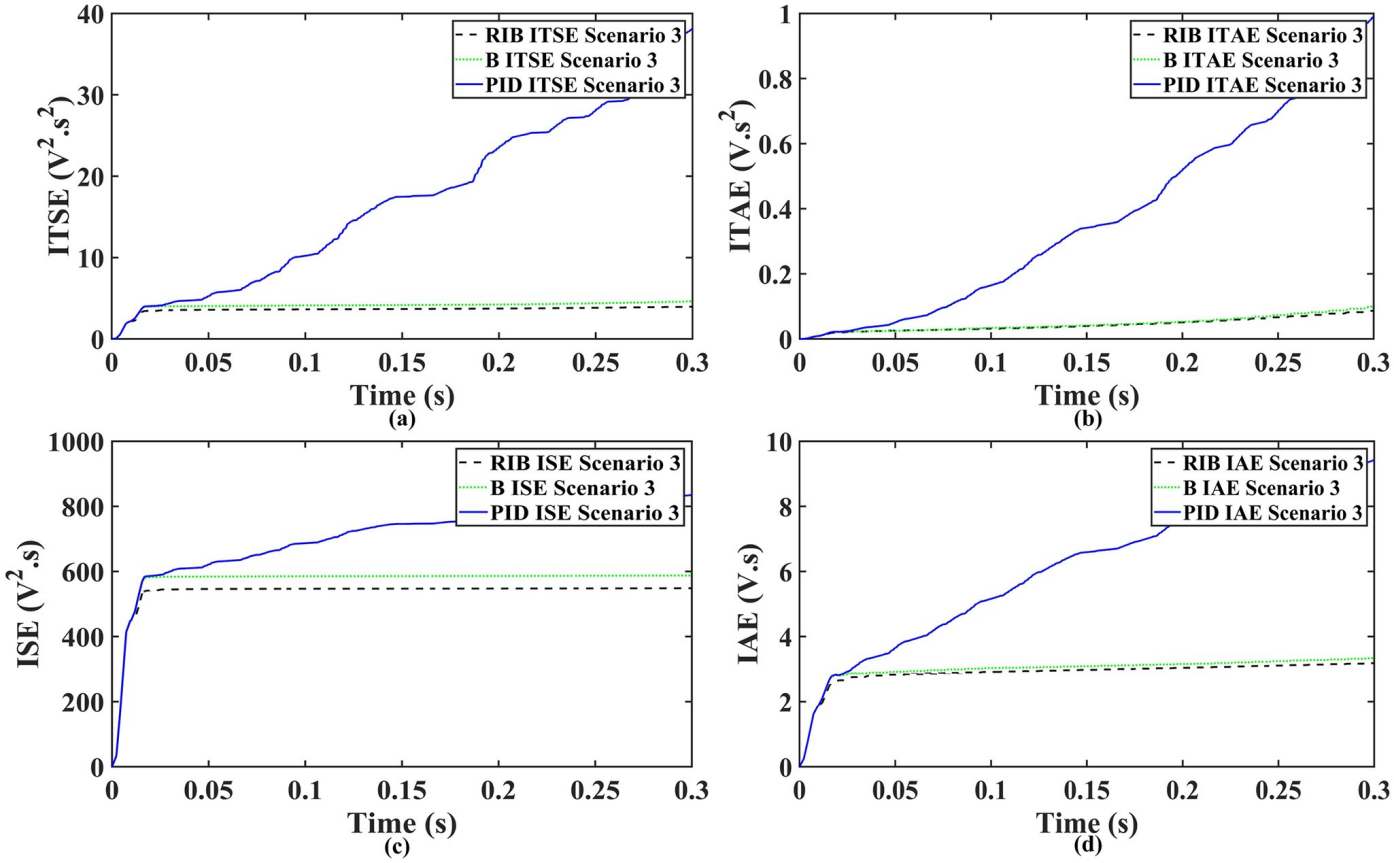
Performance indices of inverter output voltage with resistive, inductive, and capacitive loads (a)ITSE (b)ITAE (c)ISE (d)IAE.

The resistive, inductive, and capacitive loads are employed in scenario 3. The performance of the inverter with different controllers are shown in Fig 21. This plot depicts four waveforms: the inverter’s output voltage at the terminal load *V*_*C*_ for three controllers and the sinusoidal reference signal *U*_*cref*_. This shows that the output current of the inverter does not affect the output voltage of the inverter. The results show that the output voltage perfectly tracks the reference voltage, which is a sinusoidal waveform. As can be seen from Fig 21, the initial response of PID and back-stepping controllers have overshoots and undershoots, whereas the proposed controller has readily tracked the reference voltage from the start without any overshoot and undershoot. This also implies the convergence time of the proposed controller is smaller. This means that the proposed controller tracks the reference voltage faster compared to the benchmark controllers. If the inverter input voltage is less than 700V which is the nominal voltage of the inverter input, then a drop appears at the output voltage. The distortion in the output voltage of the inverter at the beginning is due to insufficient voltage provided by the non-inverting buck-boost converter. After 0.01s, in the case of the proposed controller, the input voltage of the inverter exceeds 700V and the output voltage of the inverter begins to track the reference voltage. The output voltage frequency analysis and RMSE are compared in Tables 4 and 5, respectively. The fundamental voltage is about 295.9V, 297.2V, and 294.9V in the case of the PID controller, back-stepping controller, and proposed controller, respectively, at a 50Hz frequency, while the THD is 20.13%, 14.22%, and 13.04%. This means that the RMS magnitude of harmonic frequencies are 20.13%, 14.22%, and 13.04% of the RMS magnitude of the fundamental frequency. The RIB controller shows better harmonic distortion compared to benchmark controllers, which is less than 5% according to the IEEE standard. As is evident from Fig 22, the THD due to proposed controller converges to zero level earlier than the PID and back-stepping controllers. In other words, the proposed controller as fewer harmonics in the output of the inverter. The performance indices of the inverter output voltage is calculated in Fig 23 and are compared in Table 6. Fig 23 shows the tracking error in terms of four error metrics. As can be seen from the figures, PID controller has the largest error and the proposed controller has the minimum error. In addition, Fig 17(b) and 17(d) shows that the tracking error due to PID controller keeps on increasing and does not converge in finite time. The inverter, evaluated with the suggested controller in standalone mode, has low harmonic distortion, strong control performance, and a high-quality sinusoidal waveform, as can be concluded from Figs 21–23.

### 4.2 Transient performance of the inverter output voltage with the different load when the reference amplitude changes in step

#### 4.2.1 Scenario 4 When the reference amplitude changes in step

**Fig 24:**
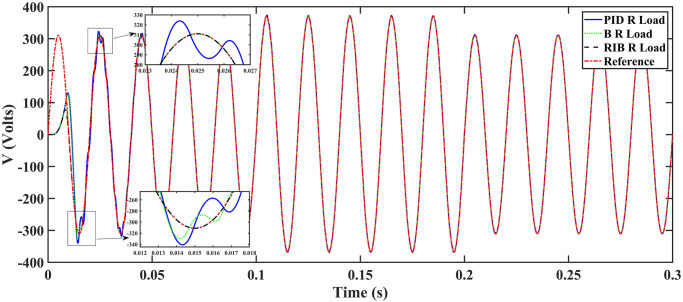
Inverter output voltage at resistive load after filtering under step changes in reference amplitude.

**Fig 25:**
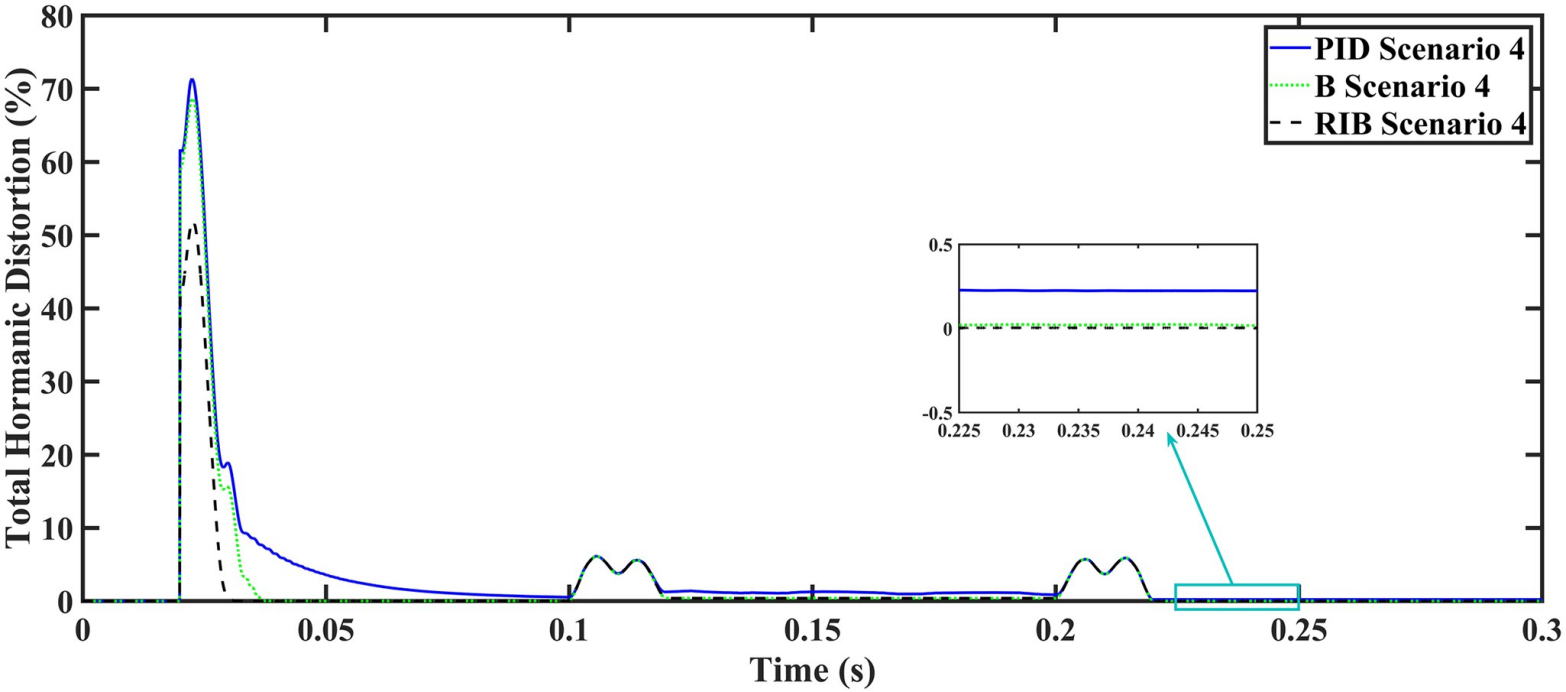
Total harmonic distortion.

**Fig 26:**
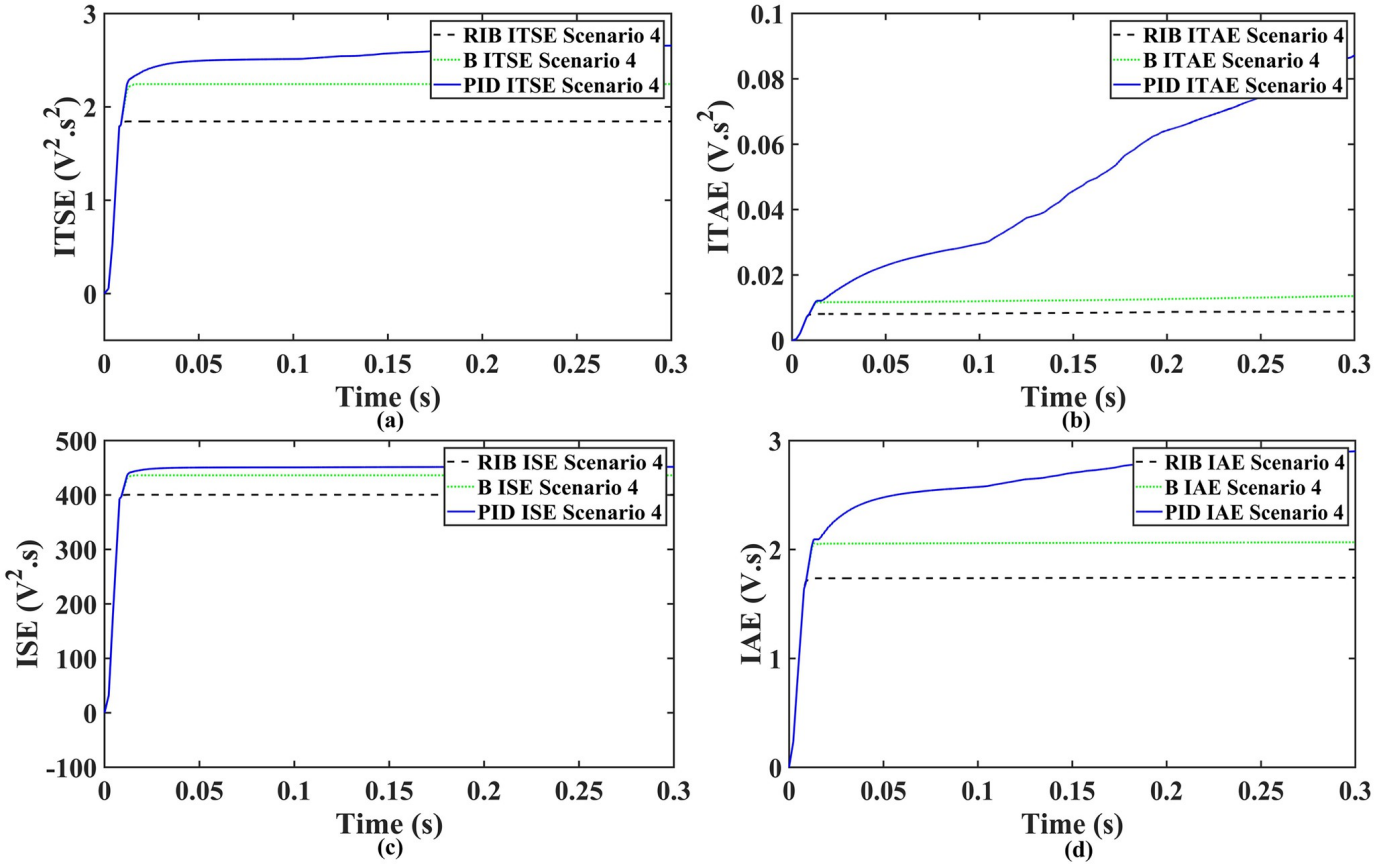
Performance indices of inverter output voltage with resistive load when the reference amplitude changes in step (a)ITSE (b)ITAE (c)ISE (d)IAE.

The MPPT results are the same as discussed in the previous section, they remain consistent for all loads. In scenario 4, a resistive load, when the reference amplitude changed in steps. The inverter’s output voltage with a resistive load under step changes in reference amplitude are displayed in Fig 24. Step changes mean that from 0.1s to 0.2s, the amplitude of the reference voltage changed from 311V to 371V and then back to 311V to check the transient performance of the system. The results show that the output voltage perfectly tracks the new reference voltage with good approximation and stability, by the proposed technique. The proposed controller tracks the reference voltage in almost 0.01s, while the back-stepping and PID controllers track it in 0.025s and 0.04s, respectively. As can be seen from Fig 24, the initial response of PID and back-stepping controllers have overshoots and undershoots, whereas the proposed controller has readily tracked the reference voltage from the start without any overshoot and undershoot. This also implies the convergence time of the proposed controller is smaller. If the inverter input voltage is less than 700V which is the nominal voltage of the inverter input, a drop appears in the output voltage. The distortion in the output voltage of the inverter at the beginning is due to the insufficient voltage provided by the non-inverting buck-boost converter. After 0.01s, in the case of the proposed controller, the input voltage of the inverter exceeds 700V and the output voltage of the inverter begins to track the reference voltage. The output voltage frequency analysis and RMSE are compared in Tables 4 and 5, respectively. The fundamental voltage is approximately 319.8V, 320.3V, and 320.8V in the case of the PID controller, back-stepping controller, and proposed controller, respectively, at a 50Hz frequency, while the THD is 14.27%, 13.82%, and 11.45%. This means that the RMS magnitude of harmonics frequencies are 14.27%, 13.82%, and 11.45% of the RMS magnitude of the fundamental frequency. The RIB controller exhibits better harmonic distortion compared to benchmark controllers, which is less than 5% according to the IEEE standard. As is evident from Fig 25, the THD due to proposed controller converges to zero level earlier than the PID and back-stepping controllers. In other words, the proposed controller as fewer harmonics in the output of the inverter. The performance indices of the inverter output voltage is calculated in Fig 26 and are compared in Table 7. Fig 26 shows the tracking error in terms of four error metrics. As can be seen from the figures, PID controller has the largest error and the proposed controller has the minimum error. In addition, Fig 17(b) and 17(d) shows that the tracking error due to PID controller keeps on increasing and does not converge in finite time. The inverter, evaluated with the suggested controller in standalone mode, demonstrates low harmonic distortion, strong control performance, and a high-quality sinusoidal waveform, as can be concluded from Figs 24–26.

**Table 7:** Comparison of ITSE, ITAE, ISE and IAE under step changes in reference amplitude.

Scenario	Scenario 1			Scenario 2			Scenario 3		
Control Techniques	PID	B	RIB	PID	B	RIB	PID	B	RIB
**ITSE**	2.656	2.244	1.844	2.807	2.251	1.855	47.59	7.064	6.044
**ITAE**	0.0872	0.0135	0.0087	0.0189	0.0142	0.0088	1.152	0.1997	0.1522
**ISE**	451.6	436.1	400.3	452.8	436.7	401	889.8	603.8	563.1
**IAE**	2.902	2.066	1.741	3.07	2.123	1.763	10.24	3.989	3.637

#### 4.2.2 Scenario 5 When the reference amplitude changes in step

**Fig 27:**
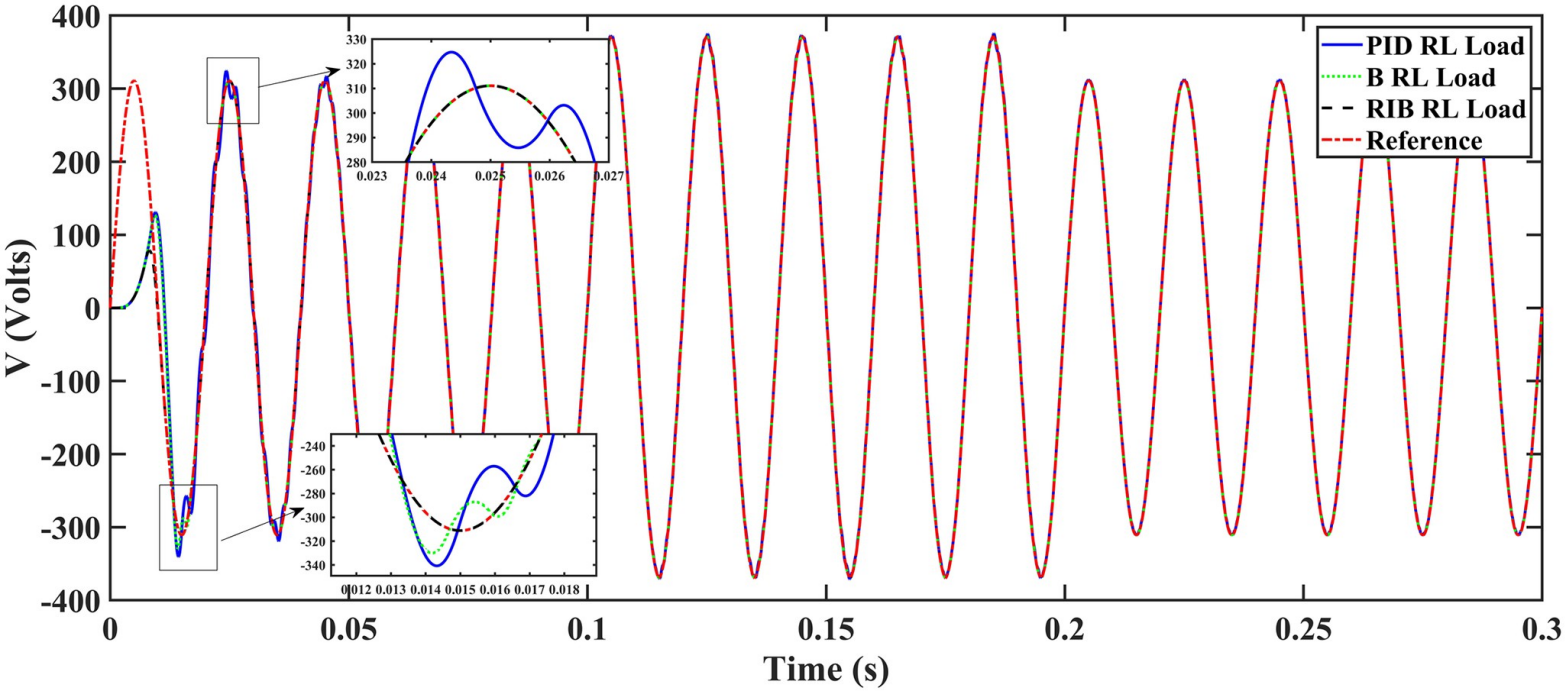
Inverter output voltage at resistive inductive loads after filtering under step changes in reference amplitude.

**Fig 28:**
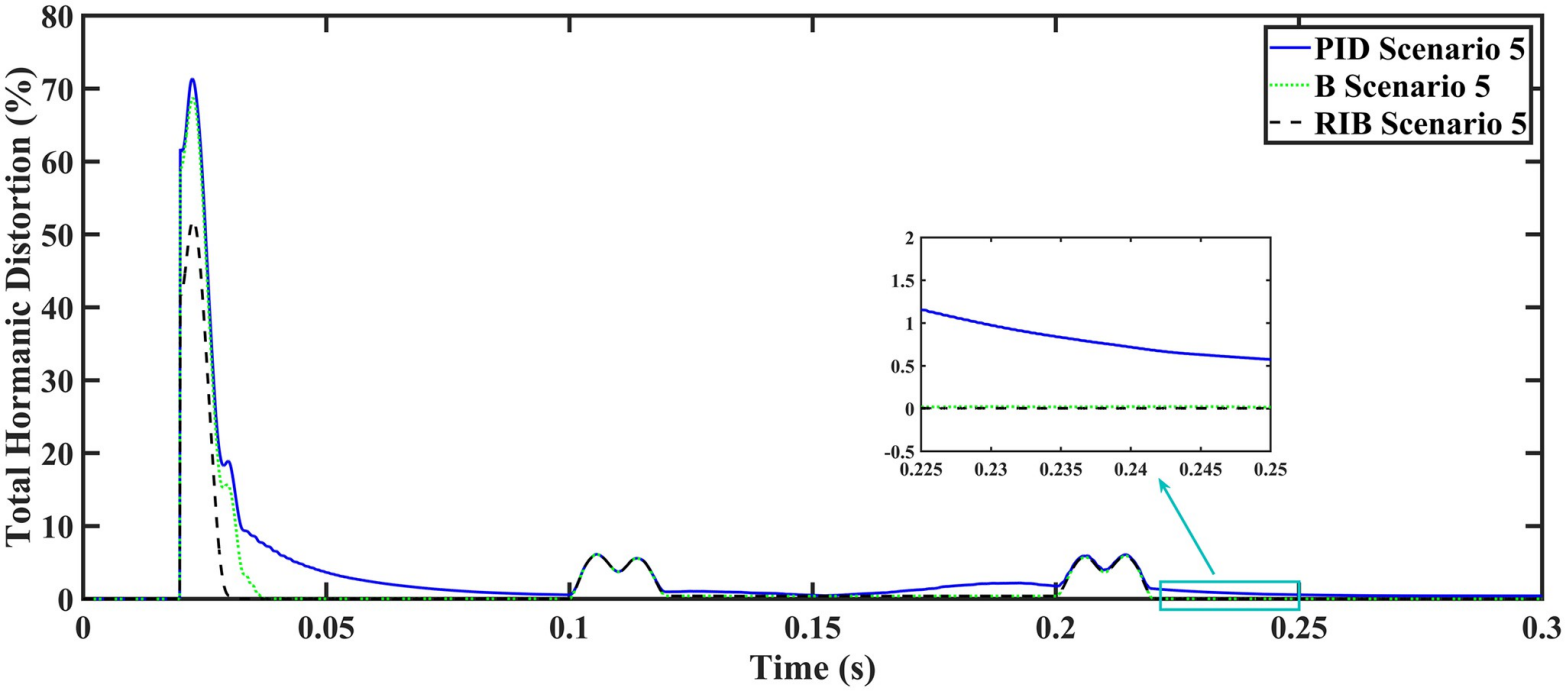
Total harmonic distortion.

**Fig 29:**
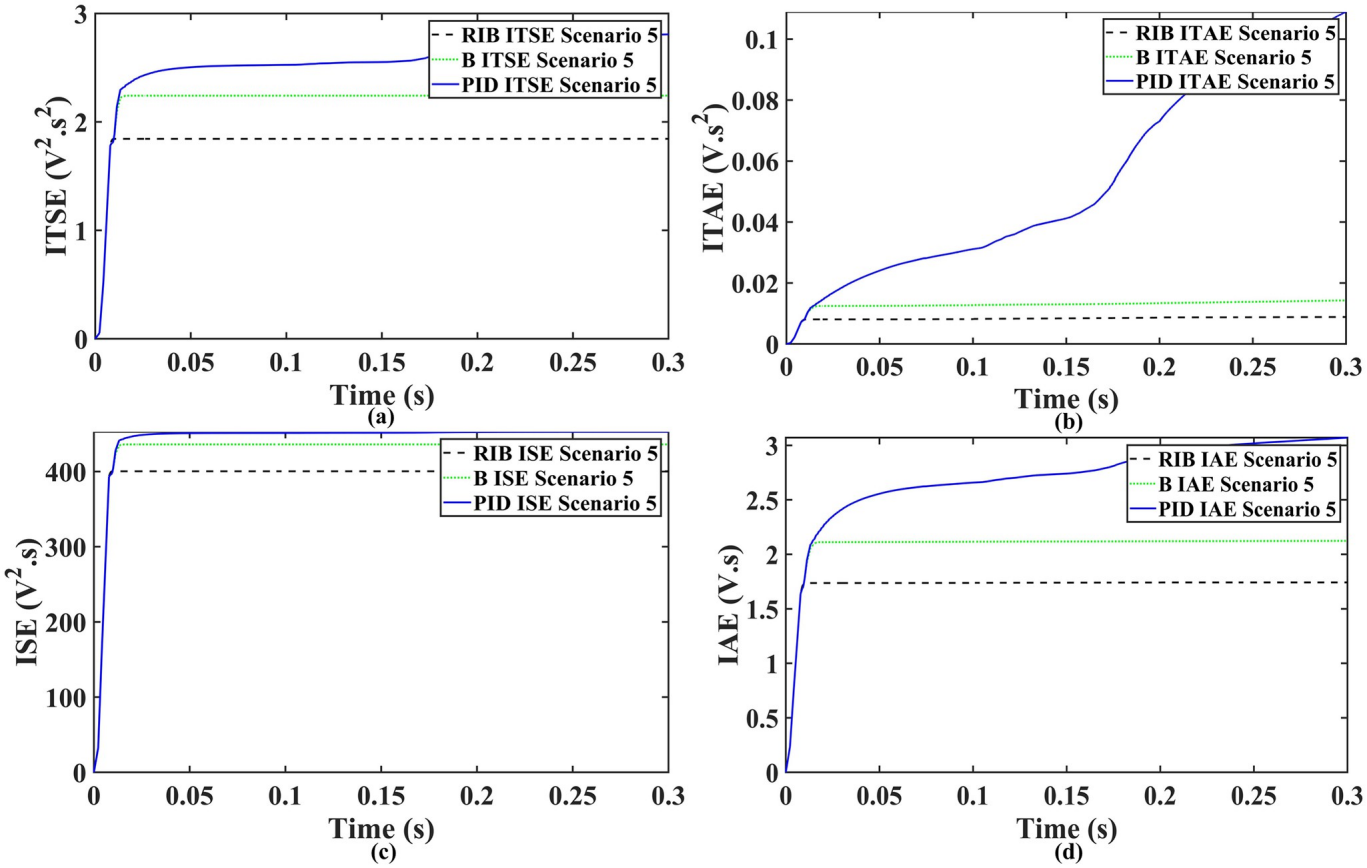
Performance indices of inverter output voltage with the resistive inductive loads when the reference amplitude changes in step (a)ITSE (b)ITAE (c)ISE (d)IAE.

We assume a resistive inductive loads in scenario 5, where the reference amplitude changes in steps. The inverter’s output voltage with a resistive inductive loads when the reference amplitude changes in steps are shown in Fig 27. Step changes mean that from 0.1s to 0.2s, the amplitude of the reference voltage changes from 311V to 371V and then back to 311V to assess the transient performance of the system. The results show that the output voltage perfectly tracks the new reference voltage with good approximation and stability, due to the proposed technique. The proposed controller tracks the reference voltage in almost 0.01s, while the back-stepping and PID controllers track it in 0.025s and 0.04s. As can be seen from Fig 27, the initial response of PID and back-stepping controllers have overshoots and undershoots, whereas the proposed controller has readily tracked the reference voltage from the start without any overshoot and undershoot. This also implies the convergence time of the proposed controller is smaller. If the inverter input voltage is less than 700V which is the nominal voltage of the inverter input, a drop appears in the output voltage. The distortion in the output voltage of the inverter at the beginning is due to insufficient voltage provided by the non-inverting buck-boost converter. After 0.01s, in the case of the proposed controller, the input voltage of the inverter exceeds 700V and the output voltage of the inverter begins to track the reference voltage. The output voltage frequency analysis and RMSE are compared in Tables 4 and 5, respectively. The fundamental voltage is approximately 319.7V, 320.3V, and 320.8V in the case of the PID controller, back-stepping controller, and proposed controller, respectively, at a 50Hz frequency, while the THD is 14.28%, 13.82%, and 11.45%. This means that the RMS magnitude of harmonic frequencies are 14.28%, 13.82%, and 11.45% of the RMS magnitude of the fundamental frequency. The RIB controller exhibits better harmonic distortion compared to benchmark controllers, which is less than 5% according to the IEEE standard. As is evident from Fig 28, the THD due to proposed controller converges to zero level earlier than the PID and back-stepping controllers. In other words, the proposed controller as fewer harmonics in the output of the inverter. The performance indices of the inverter output voltage is calculated in Fig 29 and are compared in Table 7. Fig 29 shows the tracking error in terms of four error metrics. As can be seen from the figures, PID controller has the largest error and the proposed controller has the minimum error. In addition, Fig 17(b) and 17(d) shows that the tracking error due to PID controller keeps on increasing and does not converge in finite time. The inverter, evaluated with the suggested controller in standalone mode, has low harmonic distortion, strong control performance, and a high-quality sinusoidal waveform, as can be concluded from Figs 27–29.

#### 4.2.3 Scenario 6 When the reference amplitude changes in step

**Fig 30:**
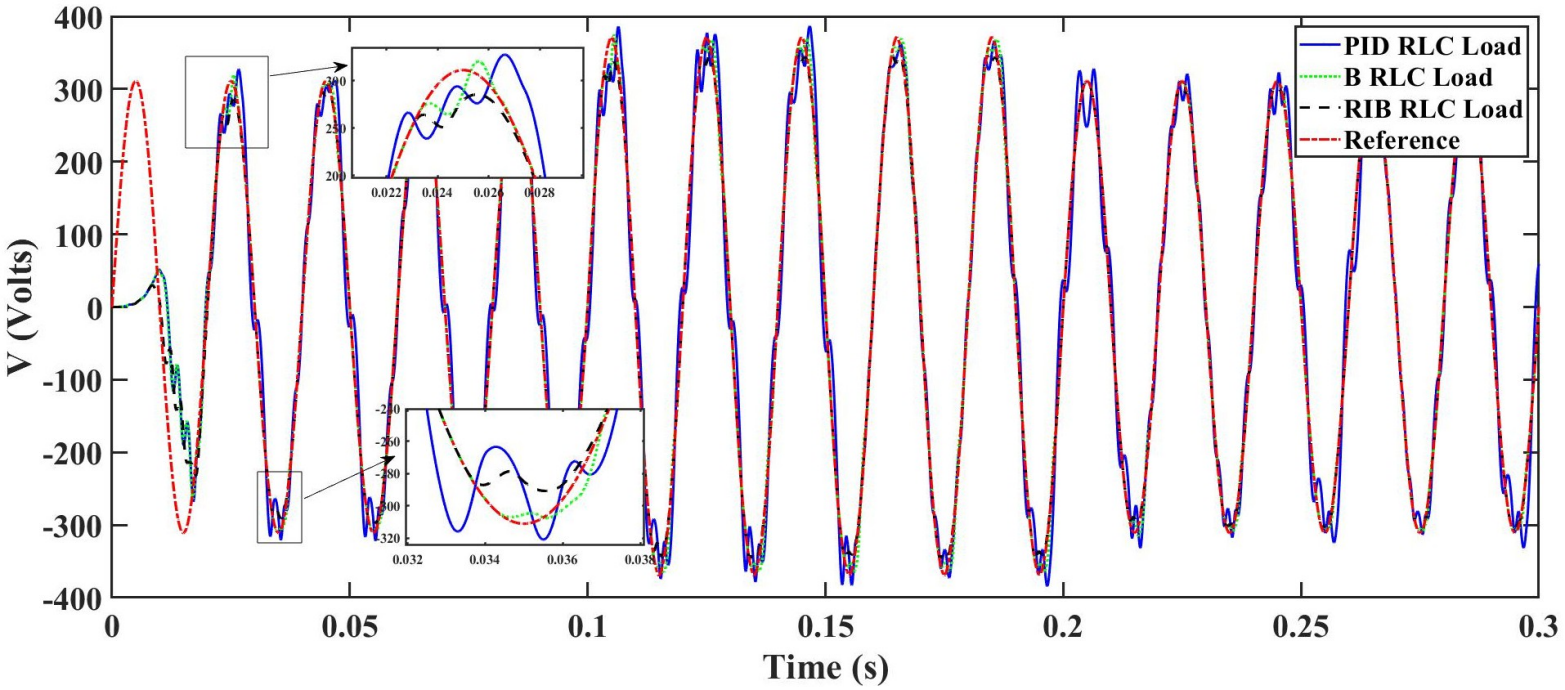
Inverter output voltage at resistive, inductive, and capacitive loads after filtering under step changes in reference amplitude.

**Fig 31:**
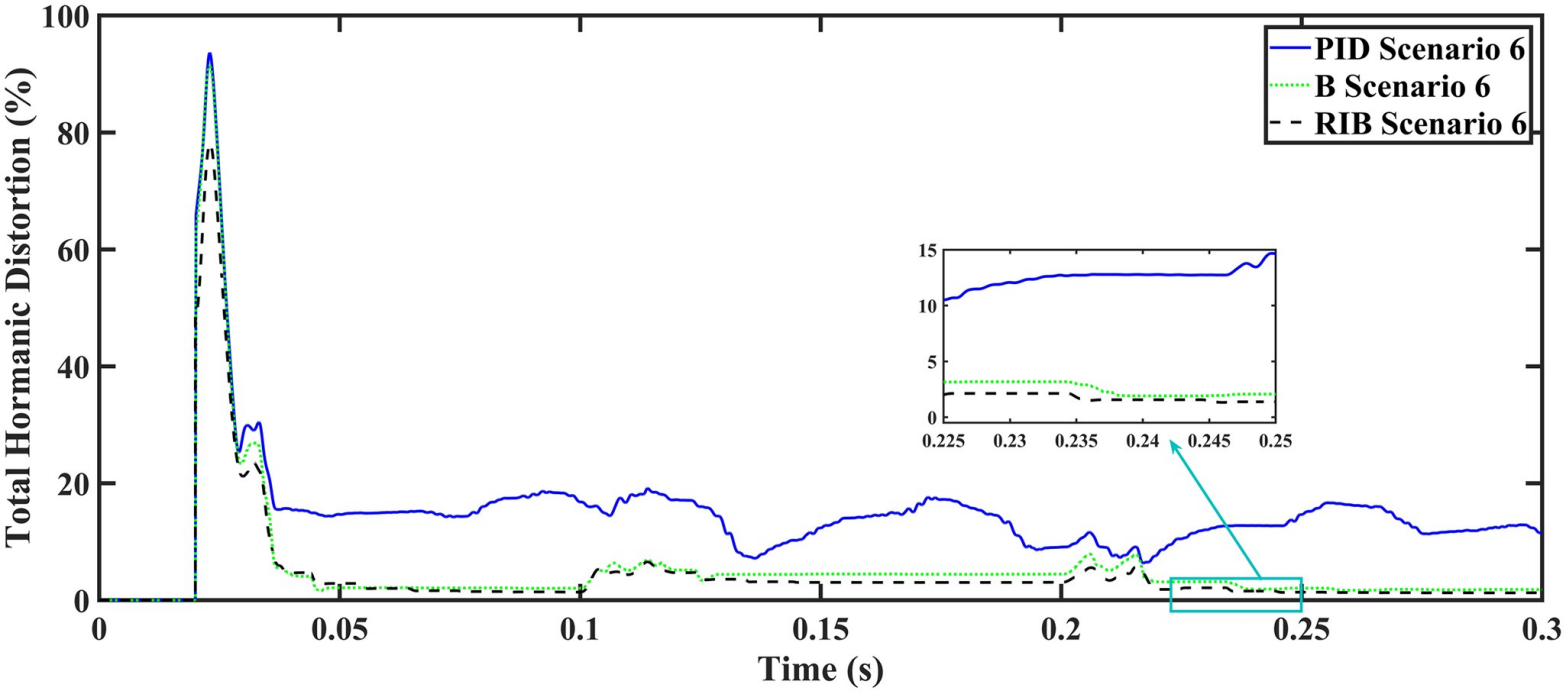
Total harmonic distortion.

**Fig 32:**
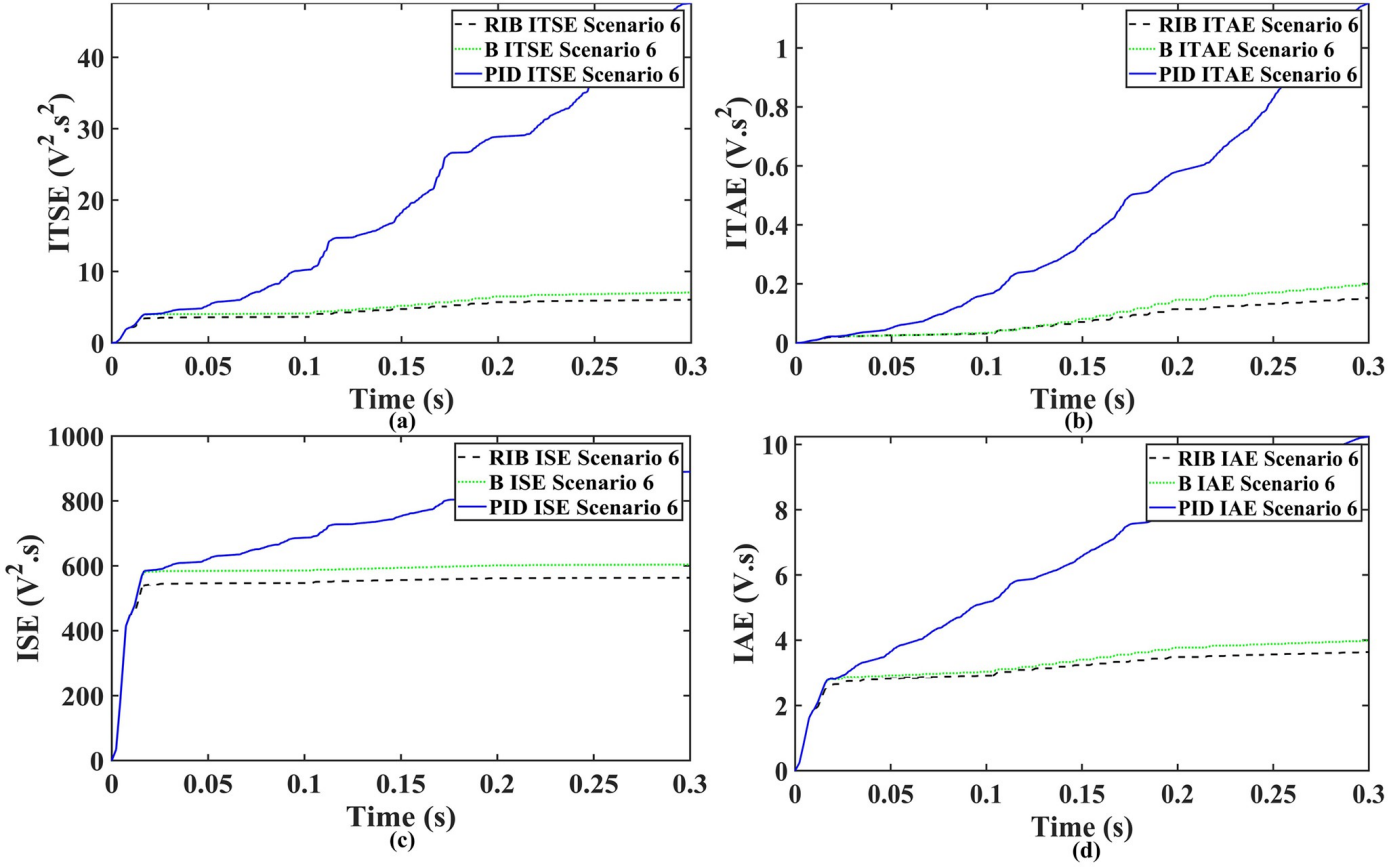
Performance indices of inverter output voltage with resistive inductive and capacitive loads when the reference amplitude changes in step (a)ITSE (b)ITAE (c)ISE (d)IAE.

A resistive, inductive, and capacitive loads is employed in scenario 6, where the reference amplitude changes in steps. The inverter’s output voltage with a resistive, inductive, and capacitive loads when the reference amplitude changes in steps is shown in Fig 30. Step changes mean that from 0.1s to 0.2s, the amplitude of the reference voltage changes from 311V to 371V and then back to 311V to assess the transient performance of the system. The results show that the output voltage perfectly tracks the new reference voltage with good approximation and stability, by the proposed technique. As can be seen from Fig 30, the initial response of PID and back-stepping controllers have overshoots and undershoots, whereas the proposed controller has readily tracked the reference voltage from the start without any overshoot and undershoot. This also implies the convergence time of the proposed controller is smaller. This means that the proposed controller tracks the reference voltage faster compared to the benchmark controller. If the inverter input voltage is less than 700V which is the nominal voltage of the inverter input, a drop appears in the output voltage. The distortion in the output voltage of the inverter at the beginning is due to insufficient voltage provided by the non-inverting buck-boost converter. After 0.01s, in the case of the proposed controller, the input voltage of the inverter exceeds 700V and the output voltage of the inverter begins to track the reference voltage. The output voltage frequency analysis and RMSE are compared in Tables 4 and 5, respectively. The fundamental voltage is approximately 314.1V, 316.4V, and 310.3V in the case of the PID controller, back-stepping controller, and proposed controller, respectively, at a 50Hz frequency, while the THD is 21.57%, 15.76%, and 14.33%. This means that the RMS magnitude of harmonic frequencies are 21.57%, 15.76%, and 14.33% of the RMS magnitude of the fundamental frequency. The RIB controller exhibits better harmonic distortion compared to benchmark controllers, which is less than 5% according to the IEEE standard. As is evident from Fig 16, the THD due to proposed controller converges to zero level earlier than the PID and back-stepping controllers. In other words, the proposed controller as fewer harmonics in the output of the inverter. The performance indices of the inverter output voltage is calculated in Fig 32 and are compared in Table 7. Fig 32 shows the tracking error in terms of four error metrics. As can be seen from the figures, PID controller has the largest error and the proposed controller has the minimum error. In addition, Fig 17(b) and 17(d) shows that the tracking error due to PID controller keeps on increasing and does not converge in finite time. The inverter, evaluated with the suggested controller in standalone mode, demonstrates low harmonic distortion, strong control performance, and a high-quality sinusoidal waveform, as can be concluded from Figs 30–32.

## 5. Conclusion

In this paper, a robust integral back-stepping nonlinear MPPT algorithm for single-phase PV inverters is designed to extract the maximum power under varying environmental conditions. The proposed techniques are analyzed in two different scenarios: 1) Steady-state performance of the output voltage of the inverter with different loads 2) Transient performance of the output voltage of inverter with different loads under step changes in reference amplitude. A reference voltage is generated through a regression plane based on the changes in environmental conditions. The simulation results demonstrate two key achievements. First, the extraction of a maximum power from the PV module using a nonlinear robust integral back-stepping controller in conjunction with a buck-boost converter. Second, the generation of sinusoidal waveform for the inverter’s output voltage, either at 311V (peak-peak) or 220V (RMS) with a fixed frequency of 50Hz. These results are assessed using the RIBSC, which effectively minimizes the convergence error. Simulation results also indicate that the proposed controller has a lower THD in comparison to both the back-stepping controller and the conventional PID controller.
